# Multiscale modeling reveals non-linear dynamics of AKT-mediated NO-ROS crosstalk in shear stress-induced endothelial phenotype

**DOI:** 10.1371/journal.pcbi.1014860

**Published:** 2026-10-21

**Authors:** Yu-Yuan Zhang, Yi-Teng Wang, Yan Qiu, Yong-Jiang Li, Dong Chen, Kai-Rong Qin

**Affiliations:** 1 Institute of Cardio-Cerebrovascular Medicine, Central Hospital of Dalian University of Technology, Dalian, Liaoning, P.R. China; 2 School of Biomedical Engineering, Faculty of Medicine, Dalian University of Technology, Dalian, Liaoning, P.R. China; Sun Yat-Sen University, CHINA

## Abstract

Endothelial dysfunction is a critical initiating event in atherosclerosis. The regulatory role of the AKT signaling pathway in the transition between injury and repair remains unclear. This study developed a multiscale model to elucidate how AKT signaling modulates endothelial repair under shear stress. The framework integrates hemodynamic stimuli, cellular behaviors, and nonlinear AKT/NO/ROS biochemical kinetics coupled with endothelial phenotypic transitions. Results demonstrate that endothelial dysfunction is driven by a spatial mismatch between NO production and ROS consumption rather than global deficiency. Phenotypic restoration is governed by the nonlinear interactions within the NO-ROS network. Systematic simulations identified a golden window, where Day 1 intervention and moderate AKT activation effectively restored endothelial cells and normalized redox status. Conversely, excessive activation caused supra-physiological over-suppression. These findings suggest that repair is determined by the tight coupling of redox homeostasis and immune dynamics, highlighting intervention timing and dosage as critical factors for precision vascular therapy.

## Author summary

Atherosclerosis, the underlying cause of many dangerous cardiovascular diseases, typically initiates with blood vessel lining damage, known as endothelial dysfunction. While the mechanical friction from flowing blood, particularly continuous disturbances, is a major driver of this injury, how cellular self-repair processes are guided by internal chemical signals remains a puzzle. To address this, we developed a comprehensive computer simulation model that connects the physical forces of blood flow with the intricate chemical interactions inside and around cells. Crucially, our model demonstrates that vessel damage is not caused by a simple lack of protective molecules like nitric oxide, but rather by a spatial mismatch where these protective signals are overwhelmed by localized bursts of destructive oxidative molecules. By simulating therapeutic interventions, we discovered a golden window for healing: turning on a key healing pathway both early and in moderation optimal for guiding damaged cells back to health. Conversely, waiting too long or over-activating the pathway could disrupt the delicate immune and chemical balance. These simulation-derived insights provide a distinct blueprint for designing smarter, precisely timed, and properly dosed therapies to repair injured blood vessels before full-scale disease develops.

## 1. Introduction

Atherosclerosis (AS) represents the common pathophysiological basis of multiple cardiovascular diseases [1,2]. Endothelial cell (EC) dysfunction is widely recognized as a critical initiating event in the onset and progression of AS [3]. Accumulating evidence indicates that endothelial injury is not an isolated or transient phenomenon, but rather a dynamic pathological process driven by abnormal hemodynamic stimuli, inflammatory responses, and oxidative stress [3–5]. In atherosclerosis-prone regions such as vascular bifurcations and curvatures, long-term exposure to oscillatory shear stress (OSS) disrupts endothelial homeostasis, thereby initiating endothelial injury and triggering a cascade of pathophysiological alterations [4]. These alterations are characterized by aberrant endothelial phenotypic transformation, excessive secretion of pro-inflammatory mediators, enhanced recruitment of immune cells, and redox imbalance, which collectively promote the initiation and progression of atherosclerotic plaques [2,4–7]. Therefore, systematic elucidation of OSS-mediated endothelial injury and its regulatory mechanisms of repair is essential for understanding AS pathogenesis and for developing effective preventive and therapeutic strategies.

Under abnormal shear stress conditions, vascular ECs undergo phenotypic transformation from a quiescent state (quiescent endothelial cells, QECs) to an activated phenotype (activated endothelial cells, AECs), followed by apoptosis or detachment upon sustained stimulation [4,8]. This phenotypic transition is accompanied by the secretion of chemokines such as monocyte chemoattractant protein-1 (MCP-1), which mediates directed recruitment of circulating monocytes [9,10]. Recruited monocytes subsequently differentiate and polarize into M1 or M2 macrophages, establishing a dynamic inflammatory microenvironment within the vascular wall [11,12]. Concurrently, both AECs and pro-inflammatory immune cells serve as major sources of reactive oxygen species (ROS) [13,14]. Persistent ROS accumulation exacerbates endothelial dysfunction and amplifies inflammatory signaling, forming a self-sustaining positive feedback loop that accelerates endothelial injury [15,16]. Collectively, this hemodynamics-driven phenotypic evolution, together with the mutual reinforcement between inflammation and oxidative stress, constitutes a central pathological mechanism underlying the initiation and progression of atherosclerosis.

During endothelial mechanochemical transduction in response to wall shear stress (WSS), the PI3K/AKT/eNOS signaling pathway functions as a key regulatory axis for maintaining vascular homeostasis [17–20]. WSS is sensed by endothelial mechanoreceptors and mechanotransduction complexes, leading to activation of AKT. Activated AKT subsequently promotes nitric oxide (NO) production through phosphorylation of endothelial nitric oxide synthase (eNOS). Beyond its vasodilatory effects, NO plays a crucial protective role by suppressing endothelial inflammation, attenuating oxidative stress, and preserving endothelial phenotypic stability [21–24]. Crucially, physiologically normal shear stress (i.e., laminar shear stress) leverages this axis to govern endothelial repair by accelerating endothelial cell proliferation, migration, and the assembly of cell-cell junctions, thereby maintaining endothelial barrier integrity [25,26]. However, under OSS conditions, the sustained phosphorylation of AKT at specific functional sites is markedly impaired, resulting in reduced NO bioavailability and a subsequent arrest of the endogenous re-endothelialization program [27]. Simultaneously, enhanced ROS-NO interactions accelerate NO depletion, ultimately leading to failure of endothelial protective and regenerative mechanisms. Consequently, AKT activity and its temporal regulation emerge as critical determinants of endothelial injury progression and repair outcomes.

Although extensive in *vivo* and in *vitro* studies have demonstrated the critical roles of WSS, AKT signaling, and inflammatory mediators in regulating endothelial function, traditional experimental approaches remain limited in capturing the complex and dynamic nature of endothelial injury and repair processes. In particular, these approaches face challenges in simultaneously resolving endothelial phenotype transitions, signaling activation kinetics, and the coupled diffusion and interaction of biochemical factors within a spatially heterogeneous microenvironment. Multiscale computational modeling provides a powerful framework to overcome these limitations by integrating processes across spatial and temporal scales, thereby enabling mechanistic interpretation of biological phenomena and prediction of emergent system behaviors.

Several multiscale modeling frameworks have been developed to investigate vascular remodeling and atherosclerotic progression. Corti et al. established a fully coupled computational fluid dynamics (CFD)-agent-based model (CFD-ABM), wherein CFD simulations are implemented to numerically solve fluid flow and quantify localized hemodynamic parameters, to reproduce vascular wall remodeling and lipid plaque formation under pathological conditions [28]. Chen et al. further applied this coupled approach to elucidate interactions between macroscale low-WSS loading and microscale cellular evolution [29]. Caballero et al. developed a hybrid framework integrating CFD, mass transport, and ABM to simulate concentric and eccentric plaque growth in coronary arteries [30]. Pichardo-Almarza and Diaz-Zuccarini constructed a quantitative systems pharmacology model to investigate the effects of simvastatin treatment on plaque growth rates [31]. Pan et al. proposed a multiscale model spanning subcellular to microenvironmental scales to predict optimal nanoparticle-based drug dosing strategies for plaque regression [32]. Concurrently, systems biology and mechanobiological models have attempted to quantify the intracellular kinetics of WSS-induced signaling, capturing the dynamic phosphorylation of Akt and eNOS activation at the sub-cellular level [33–35]. In our previous work, we also established a baseline multiscale hybrid model to simulate early-stage endothelial-leukocyte interactions driven by sustained low shear stress [36]. However, that model simplified the endothelial phenotypic switching as a unidirectional path driven solely by WSS-regulated NO depletion, leaving the antagonistic clearance of NO by ROS and the active cellular de-activation (repair) mechanisms unaddressed [36]. Despite these advances, existing modeling frameworks rarely couple the intracellular AKT-eNOS signaling kinetics with both oxidative stress and the dynamic, reversible homeostasis of endothelial repair within a spatially heterogeneous microenvironment.

In this study, a multiscale computational model was extended from our validated multi-scale cellular Potts framework [36] to investigate the spatiotemporal regulation of endothelial injury and repair under OSS conditions. To capture the continuous and coupled processes of endothelial dysfunction and its subsequent recovery, we explicitly integrated an intracellular AKT/eNOS/NO/ROS signaling network into the cellular behavioral rules. Specifically, our extended framework accounts for the mutual reinforcement between inflammation and oxidative stress (ROS), while evaluating how AKT-mediated signaling promotes endothelial restoration (reversion from the activated to the quiescent state) and suppresses pro-inflammatory cascades. Within a unified platform, the model integrates spatial heterogeneity of WSS, endothelial phenotypic transitions, monocyte recruitment, and macrophage polarization. Using this model, the effects of AKT-mediated signaling regulation on endothelial repair outcomes were systematically examined under different intervention timings and activation intensities. This study addresses key limitations of existing models in describing spatial heterogeneity and repair dynamics of endothelial dysfunction, provides a distinct, repair-centric mechanical insight into early atherogenesis, and offers a theoretical basis for precise target optimization in AKT-mediated anti-inflammatory therapies.

## 2. Methods

### 2.1. Overall multi-scale model architecture and inheritance

We extended our previously validated multiscale cellular Potts modeling (CPM) framework to investigate the spatiotemporal regulation of endothelial injury and active phenotypic restoration under highly atherogenic, OSS conditions. The integrated hybrid architecture bridges three distinct hierarchical scales through a tightly coupled ODE-PDE-CPM framework implemented on the CompuCell3D (CC3D) platform (v4.6.2), as schematically illustrated in Fig 1. At the molecular scale, intracellular signaling kinetics are solved at a second-level resolution utilizing a system of ordinary differential equations (ODEs). At the tissue scale, extracellular biochemical transport fields are governed by partial differential equations (PDEs) to simulate diffusion and reaction kinetics across the microenvironment. Finally, at the cellular scale, dynamic cell-cell interactions, membrane fluctuations, and phenotypic evolution are updated at a minute-level resolution via the Glazier-Graner-Hogeweg (GGH) algorithm.

**Fig 1:**
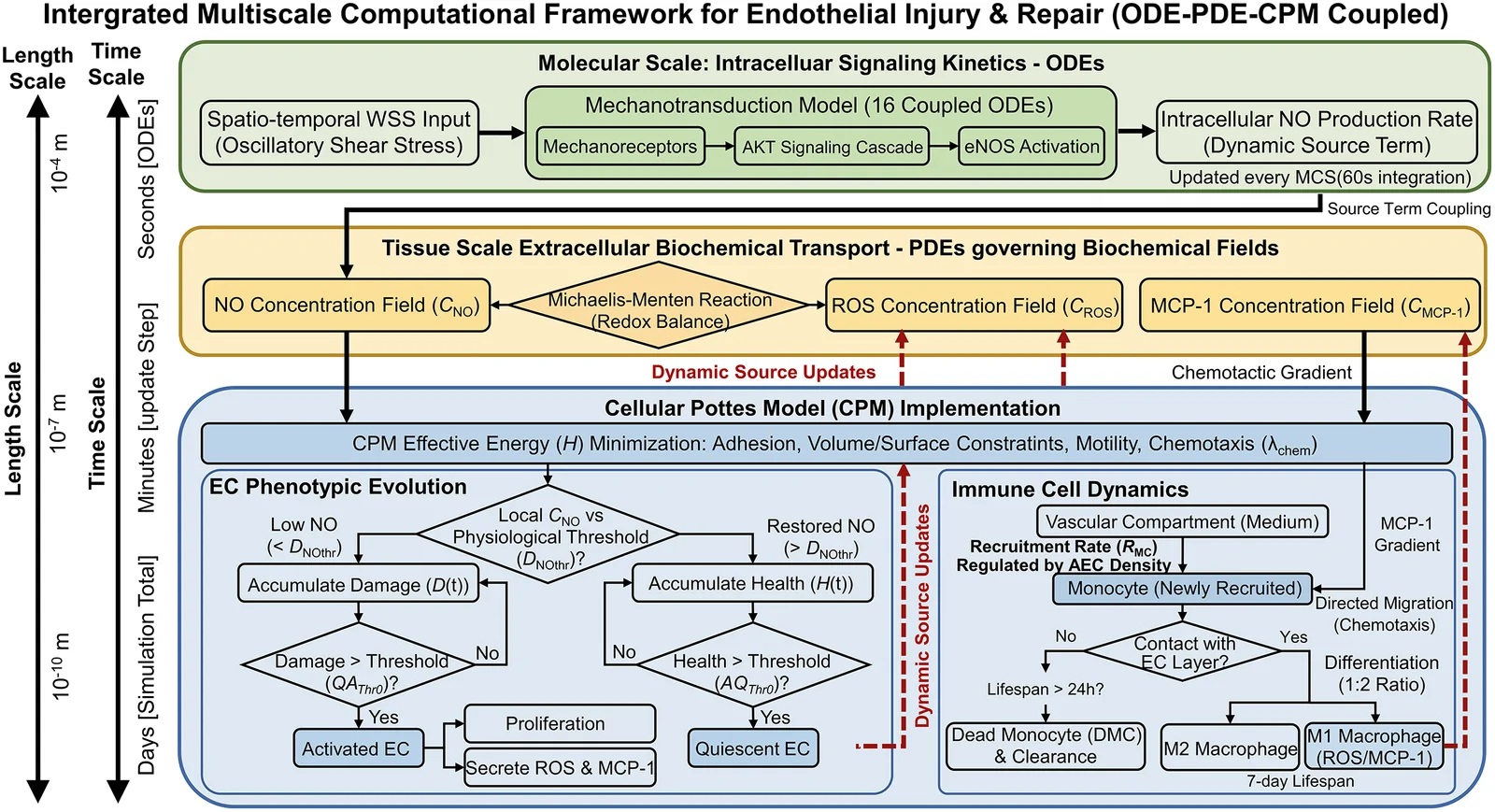
Schematic illustration of the integrated multiscale computational framework for endothelial injury and repair. The model couples ordinary differential equations (ODEs), partial differential equations (PDEs), and the cellular Potts model (CPM) to bridge molecular, cellular, and tissue length and time scales. This framework integrates mechanotransduction signaling, biochemical transport, and cellular phenotypic dynamics to simulate vascular endothelial responses to injury and the subsequent repair process.

To maintain methodological transparency and continuity, the baseline computational lattice, cell volume/surface area constraints, and fundamental Monte Carlo pixel-copy routines were inherited from our previous work [36]. However, the current framework introduces two critical, non-incremental mechanistic advancements: (1) the transition from a simplistic pulsatile flow model to a bidirectional OSS field, and (2) the integration of an extracellular ROS PDE that operates as a competitive chemical sink, directly modulating NO bioavailability.

### 2.2. Cellular Potts Model (CPM) implementation

In the CPM framework, cellular attributes, behaviors, and interactions are governed by an effective energy function *H*, which is minimized through stochastic cell membrane fluctuations. The effective energy is defined as:


$$\begin{array}{c} H=H_{con}+H_{vol}+H_{surf}+H_{chem}+H_{f} \end{array}$$ (1)

where *H*_con_ denotes the contact energy that characterizes adhesive interactions between neighboring cells of different types, thereby regulating cell-cell cohesion and segregation; *H*_vol_ and *H*_surf_ represent the energy penalties associated with deviations from the target cell volume and surface area, respectively, ensuring mechanical stability and morphological integrity of cells; *H*_chem_ corresponds to the effective energy associated with chemotactic responses of monocytes to gradients of MCP-1, enabling directed monocyte recruitment toward injured endothelial regions.; *H*_*f*_ describes the motility constraint energy that regulates intrinsic cell movement and random membrane fluctuations, thereby controlling cell migratory activity within the tissue domain.

The individual energy components are mathematically formulated as follows:


$$\begin{array}{c} H_{con}=\sum_{i,j}J\left(\tau \left(\sigma_{i}\right),\tau \left(\sigma_{j}\right)\right)(1-\delta (\sigma_{i},\sigma_{j})) \end{array}$$ (2)

$$\begin{array}{c} H_{vol}=\sum_{\sigma }\lambda_{vol}(v_{\sigma }-V_{\sigma }{)}^{\text{2}} \end{array}$$ (3)

$$\begin{array}{c} H_{surf}=\sum_{\sigma }\lambda_{surf}(s_{\sigma }-S_{\sigma }{)}^{\text{2}} \end{array}$$ (4)

$$\begin{array}{c} H_{chem}=-\lambda_{chem}(c_{j}-c_{i}{)}^{\text{2}} \end{array}$$ (5)

$$\begin{array}{c} H_{f}=-\lambda_{f}(p_{j}-p_{i}{)}^{\text{2}} \end{array}$$ (6)

Where *τ* denotes the cell type; *σ* denotes the unique cell identifier; *i* and *j* represent neighboring lattice sites; *J* represents the contact energy parameter between two neighboring cells; *J*(*τ*(*σ*_*i*_), *τ*(*σ*_*j*_)) is the contact energy parameter characterizing adhesive interactions between two adjacent cells of types *τ*(*σ*_*i*_) and *τ*(*σ*_*j*_);δ (*σ*_*i*_, *σ*_*j*_) the Kronecker delta function, which equals 1 when *σ*_*i*_ = *σ*_*j*_ and 0 otherwise. The volume constraint coefficient *λ*_vol_ penalizes deviations of the current cell volume *v*_*σ*_ from the prescribed target volume *V*_*σ*_, and the surface area constraint coefficient *λ*_surf_ enforces deviations of the current surface area *s*_*σ*_ from the target surface area *S*_*σ*_, with the magnitude of the *λ*_vol_ and *λ*_surf_ value determines the deformability of the cell. The chemotaxis coefficient *λ*_chem_ governs the strength of chemotactic responses, where *c*_*i*_ and *c*_*j*_ denote the concentrations of the MCP-1 at the source and target lattice sites, respectively, evaluated at the time of a copy attempt. The motility constraint coefficient *λ*_*f*_ defines the magnitude and direction of active cell movement, and *p*_*i*_ and *p*_*j*_ represent the position vectors of the source and target lattice sites, respectively, during the copy attempt.

Cell dynamics within the CPM framework are driven by a stochastic modified Metropolis algorithm consisting of a series of pixel-copy attempts. During each copy attempt, a source pixel *i* is randomly selected from the lattice, followed by random selection of an adjacent target pixel *j*. If the source and target pixels belonged to different cells identifiers (*σ*_*i*_ ≠ *σ*_*j*_), a copy event is attempted to overwrite the state of the target pixel with that of the source pixel. Conversely, if both pixels belong to the same cell, the copy attempt is immediately rejected. The acceptance probability, *P* (*σ*_*i*_→ *σ*_*j*_), a valid copy attempt is determined by the net change in effective energy, Δ*H*, according to the Boltzmann-style probability distribution:


$$\begin{array}{c} P(\sigma_{i}\to \sigma_{j})=\left\{ \begin{array}{c} @lc@exp(-\frac{\Delta H}{T_{m}}text,\;\Delta H>0\\ \text{1,}\;\;\;\Delta H\leq 0 \end{array}\right. \end{array}$$ (7)

Where *T*_*m*_ denotes the effective membrane fluctuation temperature, which controls the amplitude of intrinsic stochastic cell motility. This acceptance rule inherently favors configuration changes that decrease the total effective energy of the system, while simultaneously permitting energetically unfavorable transitions with a finite probability, thereby preventing the computational system from becoming trapped in local energy minima. To ensure unbiased spatial sampling, the total number of copy attempts per Monte Carlo Step (MCS) is set proportional to the total number of lattice pixels across the computational domain.

### 2.3. Subcellular signaling pathways and hemodynamic boundary conditions

To capture molecular mechanotransduction, an intracellular endothelial signaling module consisting of 16 coupled ODEs is embedded within each live EC agent. This baseline kinetic module, originally developed by Sriram et al. [35] and validated in our group. The module describes shear stress-induced AKT activation, which is associated with phosphorylation at Thr308 and Ser473 [37]. Activated AKT then promotes eNOS phosphorylation at Ser1177 and NO production. These signaling events are initiated by mechanoreceptors, including integrins, GPCRs, and ion channels, under different shear-flow conditions [38]. All downstream intracellular biochemical reactions are modeled using standard mass-action laws and Michaelis-Menten formulations (see S1 Text for the detailed ODEs and parameter values).

To bypass the severe computational inefficiencies of conventional forward/backward Euler or Runge-Kutta solvers when simultaneously integrating thousands of spatial cell coordinates, we adopted a Partially Analytical Linearization-Based ODE Solver (PALOS). Inherited from our previous methodological framework [36], PALOS drastically reduces computation times while maintaining strict numerical parity with conventional numerical frameworks; a rigorous benchmarking comparison verifying the accuracy and efficiency of PALOS under OSS conditions is provided in the S1 Fig.

The simulated spatial domain consists of a two-dimensional arterial tissue slice measuring 400 × 100 × 1 pixels, where each pixel side represents a physical length of 2 μm, translating into a tissue domain of 0.8 mm × 0.2 mm. Treating blood as a Newtonian viscous fluid, the velocity gradient along the narrow transverse (*y*) axis is considered negligible, yielding a constant shear stress distribution across the vessel width [36]. In the present model, the blood-flow velocity field and flow direction are not explicitly solved. Instead, WSS is prescribed as an external mechanical input at each spatial position to investigate the spatially dependent endothelial responses to different local shear-stress conditions. Accordingly, to reproduce the spatially heterogeneous hemodynamic environment in atheroprone regions, such as coronary bifurcations and bends, the baseline WSS magnitude, *τ*(*x*,*y*), is prescribed as a symmetric V-shaped spatial profile along the longitudinal (*x*) axis as follows [36]:


$$\begin{array}{c} \tau \left(x,y\right)=\tau_{min}+(\tau_{max}-\tau_{min}frac\left|x-x_{\text{half-width}}\right|x_{\text{half-width}},x\in \left[0,400\right],y\in \left[0,100\right] \end{array}$$ (8)

Where *τ*(*x*,*y*) denotes the baseline WSS magnitude at spatial position (*x*,*y*). *τ*_min_ = 5 dyn/cm^2^ and *τ*_max_ = 15 dyn/cm^2^ represent the minimum and maximum baseline WSS magnitudes, respectively, and *x*_half-width_ = 400 μm defines the center of the low-shear region*.*

To introduce periodic flow reversal and reproduce oscillatory shear conditions, the instantaneous OSS is defined as [33]:


$$\begin{array}{c} \tau_{\text{oss}}\text{(}x,y,t\text{)}=\tau \left(x,y\right)cos\left(2\pi ft\right) \end{array}$$ (9)

Where *τ*_oss_(*x*, *y*, *t*) denotes the instantaneous OSS at spatial coordinates (*x*, *y*) and time *t*. The spatially varying baseline WSS magnitude *τ*(*x*,*y*) determines the local OSS amplitude. The local oscillation amplitude ranges from 5 to 15 dyn/cm^2^ across the computational domain. *f* is the oscillation frequency, and *t* denotes time. The cosine term causes periodic changes in the sign of WSS, thereby representing bidirectional flow reversal. The temporal oscillation of WSS represents the dynamic mechanical stimulus applied to endothelial cells, rather than an explicitly resolved blood-flow vector field. This local instantaneous OSS serves as the continuous mechanical input to the intracellular 16-ODE signaling module at each simulation iteration.

### 2.4. Spatiotemporal transport and non-linear NO-ROS interactions

Unlike previous baseline frameworks that simplified NO depletion as a passive independent decay process, the current model explicitly tracks the extracellular redox status under OSS. The spatial transport, continuous production, metabolic degradation, and reciprocal chemical neutralization of the NO and ROS fields are solved using coupled reaction-diffusion PDEs via the *DiffusionSolverFE* module in CC3D:


$$\begin{array}{c} \frac{\partial C_{\text{NO}}}{\partial t}=\underset{\text{diffusion}}{\underbrace{D_{\text{NO}}\nabla^{\text{2}}C_{\text{NO}}}}+\underset{\text{production}}{\underbrace{k_{\text{pQNO}}N_{\text{QEC}}+k_{\text{pANO}}N_{\text{AEC}}}}-\underset{\text{decay}}{\underbrace{k_{\text{dNO}}C_{\text{NO}}}}-\underset{\text{reaction}}{\underbrace{\frac{V_{\text{NO-ROS}}^{max}C_{\text{NO}}C_{\text{ROS}}}{K_{\text{NO-ROS}}+C_{\text{ROS}}}}} \end{array}$$ (10)

$$\begin{array}{c} \begin{array}{c} \frac{\partial C_{\text{ROS}}}{\partial t}=\underset{\text{diffusion}}{\underbrace{D_{\text{ROS}}\nabla^{\text{2}}C_{\text{ROS}}}}+\underset{\text{production}}{\underbrace{k_{\text{pROS}}N_{\text{AEC}}(1+\frac{D_{\text{currenrt}}}{D_{\text{currenrt}}\text{+ADThr0}})+k_{\text{pROS}}N_{\text{M1}}}}-\underset{\text{decay}}{\underbrace{k_{\text{dROS}}C_{\text{ROS}}}}\mathrm{\backslash hfill}\\ -\underset{\text{reaction}}{\underbrace{\frac{V_{\text{NO-ROS}}^{max}C_{\text{NO}}C_{\text{ROS}}}{K_{\text{NO-ROS}}+C_{\text{ROS}}}}}\mathrm{\backslash hfill} \end{array} \end{array}$$ (11)

Where *C*_NO_ and *C*_ROS_ denote the extracellular NO and ROS concentrations, respectively; *D*_NO_ and *D*_ROS_ are tissue diffusion constants; *k*_dNO_ and *k*_dROS_ represent independent metabolic decay constants. In the present model, activated endothelial cells and infiltrating pro-inflammatory M1 macrophages are explicitly considered as the cellular sources contributing to the extracellular ROS field [16,39], where *k*_pROS_ represents the basal cell-specific generation rate. This assumption is based on the established oxidative activity of activated/dysfunctional endothelial cells and pro-inflammatory macrophages and reflects the cell populations explicitly represented in the current model. It does not imply that AECs and M1 macrophages are the only ROS-producing cell types in *vivo*. The *N*_AEC_ and *N*_M1_ denote the instantaneous numbers of activated endothelial cells and M1 macrophages, respectively. The final term in both partial differential equations introduces a non-linear Michaelis-Menten formulation describing the rapid, direct chemical interaction between NO and ROS, thereby representing ROS-mediated consumption of bioavailable NO and the formation of peroxynitrite.

Critically, the damage-dependent scaling multiplier 1 + (*D*_current_ / (*D*_current_+ADThr0)) coupled to the AEC population (*N*_AEC_) does not introduce mathematical redundancy or collinearity artifacts. errors. While the integer agent count *N*_AEC_ serves as a macro-scale spatial quantification of the activated cell footprint across the grid, the continuous variable *D*_current_ explicitly tracks the micro-scale pathological severity and functional degradation within individual cell agents over time. Biologically, newly activated endothelial cells are assumed to generate ROS at a basal rate (*k*_pROS_). As endothelial dysfunction progresses under sustained mechanical and inflammatory stimulation, ROS production is progressively enhanced, representing increased oxidative activity associated with NADPH oxidases and mitochondrial dysfunction [40,41] This intracellular functional worsening drives an accelerated, non-linear oxidative burst within heavily damaged cells, a physiological transition captured mathematically by the continuous scaling factor.

Concurrently, the extracellular NO source terms (*k*_pQNO_ for QECs and *k*_pANO_ for AECs) are not treated as constants but are dynamically updated to reflect spatial heterogeneous endothelial synthesis in response to local WSS. During each MCS, the embedded ODE module calculates the instantaneous intracellular NO production rate based on the local WSS magnitude, which is subsequently released into the extracellular field as a localized source term. Endothelial cells possess distinct NO production capacities across different phenotypic states: compared with QECs, the baseline NO production rate of AECs is approximately 0.6-fold [36,42]. When a QEC transitions to the activated phenotype, the temporal decline of its NO production capacity is mathematically formulated as [36]:


$$\begin{array}{c} k_{\text{pANO}}=\left\{ \begin{array}{c} @lc@0.6k_{\text{pANO}}+0.4k_{\text{pANO}}e^{-k_{\text{dANO}}t_{\text{c}}}\text{, }k_{\text{pANO}}>0.6k_{\text{pANO}}\\ 0.6k_{\text{pANO}}\text{, }k_{\text{pANO}}\leq 0.6k_{\text{pANO}} \end{array}\right. \end{array}$$ (12)

Where *k*_dANO_ denotes the decay constant controlling the loss of NO production capacity, and *t*_c_ represents the time elapsed since the onset of EC activation. Conversely, when an AEC successfully reverts to the quiescent phenotype, its NO production capacity is linearly restored to 1/0.6 times the production rate of the activated state, successfully establishing a closed-loop, multiscale feedback framework for endothelial injury and repair.

### 2.5. Endothelial cell phenotypes transition rules

In this model, ECs are abstracted into three functional states: QECs, AECs, and DECs. Their state transitions were collectively regulated by the local NO environment and were described by Eqs (13–16) [36]. The model assumed that NO maintains endothelial homeostasis under physiological conditions, and a physiological NO reference level, *D*_NOthr_ = 0.05 μM, was introduced to characterize the directional shift between endothelial dysfunction and functional recovery.

Under abnormal WSS conditions induced by oscillatory flow, NO synthesis in QECs was suppressed. When the NO concentration at the cell centroid (*C*_cNO_) fell below the physiological reference level, a damage accumulation process (*D*(*t*)) was initiated. The damage increment was defined by Eq (13) and was accumulated over time according to Eq (14). Once the accumulated damage exceeded the predefined threshold (QAThr0), QECs were converted into the activated state, indicating the onset of endothelial dysfunction.


$$\begin{array}{c} \Delta D=\left\{ \begin{array}{c} @lc@\text{0,}\;\;\;\;\;\;\;\;\;\;\;C_{\text{cNO}}\geq D_{\text{NOthr}}\\ \frac{V_{\text{NO-}max}{C_{\text{cNO}}}^{n}}{{C_{\text{cNO}}}^{n}+{K_{\text{NO}}}^{n}}-\frac{V_{\text{NO-}max}{D_{\text{NO}}}^{n}}{{D_{\text{NO}}}^{n}+{K_{\text{NO}}}^{n}}\text{, }C_{\text{cNO}}<D_{\text{NOthr}}\; \end{array}\right. \end{array}$$ (13)

$$\begin{array}{c} D\left(t\right)=\left\{ \begin{array}{c} @lc@\text{0, }t=0\\ D(t-1)+\Delta D,\;t>0 \end{array}\right. \end{array}$$ (14)

After entering the activated state, the fate of ECs was determined by the local NO microenvironment. Under sustained low-NO conditions, AECs accumulated additional damage and were eventually converted into the dead state, followed by gradual cell shrinkage until disappearance in the simulation. In contrast, when the local NO level was restored above the physiological range, AECs began to accumulate a health variable (*H*(*t*)), as described by Eqs (15–16). Once the accumulated health exceeded the corresponding threshold (AQThr0), the cells were reversibly converted back to the quiescent state, representing the recovery of endothelial function.


$$\begin{array}{c} \Delta H=\left\{ \begin{array}{c} @lc@\frac{V_{\text{NO-}max}{C_{\text{cNO}}}^{n}}{{C_{\text{cNO}}}^{n}+{K_{\text{NO}}}^{n}}-\frac{V_{\text{NO-}max}{D_{\text{NO}}}^{n}}{{D_{\text{NO}}}^{n}+{K_{\text{NO}}}^{n}}\text{, }C_{\text{cNO}}\geq D_{\text{NOthr}}\\ 0\text{, }C_{\text{cNO}}<D_{\text{NOthr}} \end{array}\right. \end{array}$$ (15)

$$\begin{array}{c} H\left(t\right)=\left\{ \begin{array}{c} @lc@\text{0, }t=0\\ H(t-1)+\Delta H,\;t>0 \end{array}\right. \end{array}$$ (16)

Where *V*_NO-max_ denoted the maximum NO response rate, *K*_NO_ represented the Michaelis-Menten constant, and *n* was the Hill coefficient.

The model assumed that AECs possessed proliferative capability, and their cell volume increased over time, whereas the volume of QECs remained unchanged. Proliferation of AECs was considered to enhance local inflammatory responses and promote immune cell recruitment and plaque formation [8,43]. Based on in *vitro* observations that ECs completed one mitotic division approximately every 24 h, the volume growth rate of AECs (*k*_AEC_) was set to 0.01389 pixels/MCS, corresponding to a 24 h cell division cycle (Eq 17). When the current cell volume reached or exceeded twice the initial volume, mitotic division was completed.


$$\begin{array}{c} V_{\text{current}}=\left\{ \begin{array}{c} @lc@\text{25,\textbackslash{} \textbackslash{} \textbackslash{} \textbackslash{} \textbackslash{} \textbackslash{} \textbackslash{} \textbackslash{} \textbackslash{} \textbackslash{} \textbackslash{} \textbackslash{} \textbackslash{} \textbackslash{} \textbackslash{} \textbackslash{} \textbackslash{} \textbackslash{} \textbackslash{} \textbackslash{} for QECs}\\ V_{\text{previous}}+k_{\text{AEC}}\text{, for AECs} \end{array}\right. \end{array}$$ (17)

Where *V*_current_ was the volume of AECs at the current step; *V*_previous_ was the volume of AECs at the previous step; *k*_AEC_ was the growth rate of AECs.

### 2.6. Monocyte recruitment, polarization, and chemotaxis

In this model, vascular supply was assumed to be sufficient to continuously meet the demand for monocyte recruitment. The “Medium” domain was used to represent the vascular compartment. Under the baseline condition of mild endothelial injury, monocytes were recruited at a constant basal rate of one cell every 120 Monte Carlo steps (MCS) [36]. To capture the regulatory effect of endothelial injury severity on immune cell recruitment, the density of AECs was introduced as a modulation factor. The AEC density, *ρ*_AEC_(*t*), was defined at each recruitment cycle time *t* = *kT*_MC_ as the ratio of the number of AECs to the total number of viable ECs, as expressed in Eq (18). This metric was used to characterize the local degree of endothelial activation and, consequently, the overall level of vascular wall injury.


$$\begin{array}{c} \rho_{\text{AEC}}\left(t\right)=\frac{N_{\text{AEC}}\left(t\right)}{N_{\text{AEC}}\left(t\right)+N_{\text{QEC}}\left(t\right)},t=kT_{\text{MC}}(k=0,1,2,\text{\textbackslash{}ldots}) \end{array}$$ (18)

Where *N*_AEC_(*t*) denoted the number of AECs at time *t*, *N*_QEC_(*t*) denoted the number of QECs at time *t*, *T*_MC_ represented the monocyte recruitment period, and *k* was the recruitment cycle index.

On this basis, the actual monocyte recruitment rate, *R*_MC_, was defined as a piecewise function of the AEC density, as shown in Eq (19) [36]. When the AEC density was low, monocytes were recruited into the vascular compartment at the minimal level. As the AEC density increased, the recruitment rate was correspondingly elevated. Under conditions of pronounced endothelial activation, the recruitment rate reached its maximum value. Through this piecewise regulation scheme, the model was able to qualitatively capture the acceleration of immune cell recruitment induced by increasing endothelial injury without introducing additional continuous parameters.


$$\begin{array}{c} R_{\text{MC}}\left(\rho_{\text{AEC}}\left(t\right)\right)=\left\{ \begin{array}{c} @lc@3,\;\rho_{\text{AEC}}\geq 0.75\\ 2,\text{0.5}\leq \rho_{\text{AEC}}<0.75\\ 1,\;\rho_{\text{AEC}}<0.5 \end{array}\right. \end{array}$$ (19)

At each recruitment event, newly generated monocytes were randomly placed at available lattice sites within the vascular lumen. They subsequently underwent random migration within the vascular compartment. In the model, monocytes underwent three types of state transitions: differentiation, death, and clearance. When monocytes migrated to the endothelial layer and established direct contact with AECs, they were differentiated into M1 or M2 macrophages at a ratio of 2:1 [44]. Therefore, variations in M1 and M2 macrophage abundance among different AKT conditions primarily arise from changes in monocyte recruitment, rather than from AKT-dependent changes in the intrinsic polarization ratio.This differentiation influenced local endothelial function and the inflammatory microenvironment. The lifetimes of M1 and M2 macrophages were set to approximately 7 days [36,44].

In contrast, when monocytes were not recruited to the endothelial layer and remained within the vascular compartment, they were converted into dead monocytes (DMCs) after their lifespan reached the predefined limit of 24 h [45]. These DMCs then underwent gradual cell shrinkage until complete removal from the computational domain. AECs and M1 macrophages were assumed to secrete MCP-1. Monocytes migrated directionally toward the injured endothelial layer in response to the MCP-1 concentration gradient. The MCP-1 concentration field was computed using the *DiffusionSolverFE* module in the CC3D software.


$$\begin{array}{c} \frac{\partial C_{\text{MCP-1}}}{\partial t}=\underset{\text{diffusion}}{\underbrace{D_{\text{MCP-1}}\nabla^{\text{2}}C_{\text{MCP-1}}}}+\underset{\text{production}}{\underbrace{k_{\text{pAMCP-1}}N_{\text{AEC}}+k_{\text{pM1MCP-1}}N_{\text{M1}}}}-\underset{\text{decay}}{\underbrace{k_{\text{dMCP-1}}C_{\text{MCP-1}}}} \end{array}$$ (20)

Where *C*_MCP-1_ denoted the concentration field of MCP-1, *D*_MCP-1_ represented the diffusion coefficient of MCP-1, *k*_pAMCP-1_ and *k*_pM1MCP-1_ corresponded to the MCP-1 production rates of AEC and M1 macrophages, respectively, and *k*_dMCP-1_ was the decay rate of MCP-1.

### 2.7. Multiscale coupling approach and parameters

The present model employed a multiscale coupling framework that integrated ODE–based mechano-chemical signaling, PDE-based reaction-diffusion processes, and a CPM to capture the coordinated interactions across molecular, cellular, and tissue scales during endothelial injury and repair. Specifically, the ODE module was used to describe the intracellular AKT activation and its regulatory role in the recovery of NO production in ECs. The PDE module was adopted to model the reaction-diffusion dynamics of NO, ROS, and MCP-1 at the tissue scale. The CPM module was implemented to simulate cell-level behaviors, including cell migration, proliferation, volume evolution, and cell-cell adhesion. Dynamic coupling among different scales was achieved through shared state variables. The NO production rate calculated by the ODE module was incorporated into the PDE module as a source term to update the spatial-temporal NO concentration field. Meanwhile, NO reacted with ROS generated from the PDE module, and the coupled NO-ROS distributions jointly regulated EC state transitions at the cellular level. The MCP-1 concentration field served as a chemotactic signal to mediate immune cell recruitment and directed migration toward injured endothelial regions.

In terms of temporal scaling, one MCS was defined to correspond to 1 min of real time [36,46]. Considering that intracellular signaling processes occur on the order of seconds, whereas cellular and tissue-level processes evolve on the order of minutes, the ODE system was numerically integrated for 60 s during each MCS. The resulting molecular-scale outputs were iteratively passed to the PDE and CPM modules at each cellular update until the end of the simulation. The total simulation duration was set to 10,080 MCS, corresponding to 7 days of biological time [47]. All model parameters and initial molecular concentrations are summarized in Table 1. Molecular species not listed in the table were initialized with zero concentration. Quantitative results were reported as mean ± standard deviation, and each simulation scenario was independently repeated 12 times.

**Table 1:** Parameter values in this model.

Symbol	Parameter	Value	References
*τ*_min_	Minimum baseline WSS magnitude	5 dyn/cm^2^	[48]
*τ*_max_	Maximum baseline WSS magnitude	15 dyn/cm^2^	[48]
*f*	OSS frequency	1 Hz	[38]
*D*_NO_	Diffusion coefficient of NO	80 voxel^2^/MCS	[36,49]
*k*_dNO_	Decay rate of NO	0.12 /MCS	[49]
$V_{\text{NO-ROS}}^{max}$	Maximum reaction rate of NO and ROS	1 /MCS	Estimated
*K*_NO-ROS_	Michaelis-Menten constant	0.6 μM	[33]
*k*_dANO_	Constant controlling the decrease in NO production	10^-5^ /MCS	[36]
*D*_ROS_	Diffusion coefficient of ROS	80 voxel^2^/MCS	[36,49]
*k*_pROS_	Production rate of ROS	0.13 μM/cell/MCS	[33,49]
*k*_dROS_	Degradation rate of ROS	0.6 /MCS	[33]
*D*_NOthr_	Damage threshold	0.05 μM	[36]
QAThr0	Threshold of QECs transition to AECs	50 μM	Estimated
ADThr0	Threshold of AECs transition to DECs	400 μM	Estimated
AQThr0	Threshold of AECs transition to QECs	30 μM	Estimated
*V*_NO-max_	Maximum response rate of NO	0.11592 μM/MCS	[36]
*K*_NO_	Michaelis-Menten constant of NO	0.04 μM	[36]
*n*	Hill constant of NO	4	[36]
*k*_AEC_	Growth rate of AECs	0.01389 pixels/MCS	Estimated
*T*_MC_	Monocyte recruitment period	120 MCS	[36,50]
Immune cells	Lifespan of MCs	24 h	[45]
	Lifespan of M1 or M2	7 Days	[44]
	Monocyte to macrophage type ratio	M1:M2 = 2:1	[44]
*D*_MCP-1_	Diffusion coefficient of MCP-1	20.8 voxel^2^/MCS	[36]
*k*_pAMCP-1_	Production rate of MCP-1 by AECs	0.54 pg/ml/cell/MCS	[51]
*k*_pM1MCP-1_	Production rate of MCP-1 by M1	0.36 pg/ml/cell/MCS	[36]
*k*_dMCP-1_	Decay rate of MCP-1	0.12 /MCS	[51]

In the WSS-induced endothelial injury model, the ODE system governing NO production includes the AKT activation-rate parameter *k*_*af*5_, whose baseline value is provided in the S1-S2 Tables. The unit of *k*_*af*5_ is consistent with that defined in the original ODE model and is listed in the S1 Table. At the molecular scale, AKT activation was varied relative to this baseline value to characterize the responses of AKT/eNOS/NO signaling. For the subsequent multiscale endothelial-repair simulations, three representative activation strengths, 3.4, 3.8, and 4.2 *k*_*af*5_ were selected to compare weak, moderate, and strong AKT activation, respectively. The effects of different post-injury treatment initiation times were also evaluated. These results are presented and discussed in detail in the Results section.

## 3. Results

### 3.1 Construction of a Multi-scale Endothelial Injury Model

A multiscale model of endothelial injury was further developed in this study based on previous work [36]. Fig 2 illustrates the spatial distributions of cellular components and key biochemical factors within the injured microenvironment. As shown in Fig 2(a), heterogeneous spatial distributions of endothelial and immune cell populations were observed in the injured region. Under lower local OSS amplitude, QECs were activated and transitioned into AECs. Endothelial activation was associated with mitosis and migration. A fraction of AECs underwent apoptosis following detachment from the basement membrane. With progressive injury, AECs further transitioned to DECs. Meanwhile, directed recruitment of monocytes to the injured site was observed, followed by their polarization into pro-inflammatory M1 macrophages and anti-inflammatory M2 macrophages. Fig 2b-2d present the spatial concentration distributions of three key biochemical factors: MCP-1, NO, and ROS. Color gradients from blue to red indicate increasing concentrations. As shown in Fig 2(b), MCP-1 was secreted by AECs and M1 macrophages, and its concentration gradient formed a chemotactic axis that drove monocyte migration toward the injured region. Fig 2(c) shows that NO production depended on QECs and AECs under WSS stimulation. At steady state, NO production by AECs was approximately 0.6-fold that of QECs. NO was involved in the regulation of endothelial phenotypic transition. As shown in Fig 2(d), ROS was generated by AECs and M1 macrophages, and its spatial distribution reflected the level of oxidative stress in injured endothelial regions. In addition, ROS reacted with NO, which led to NO consumption and disruption of endothelial functional homeostasis. Overall, the injury model was characterized by low NO concentrations and high MCP-1 and ROS concentrations, with AECs being the dominant cellular components.

**Fig 2:**
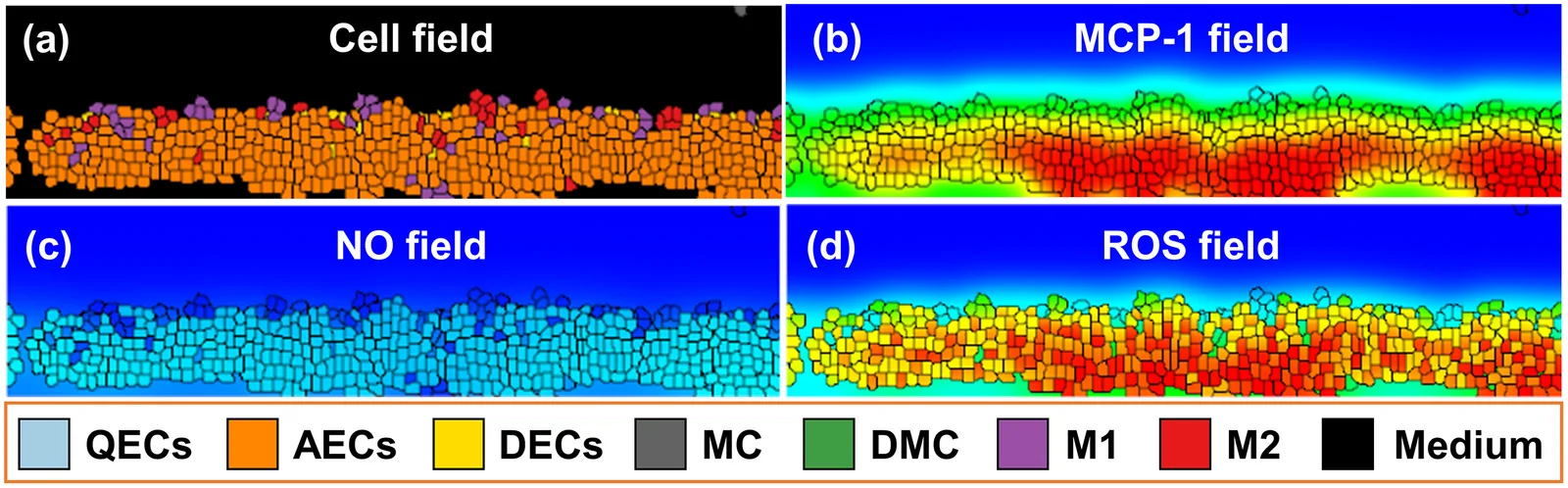
Spatial distribution of cellular components and biochemical factors in the endothelial injury model. **(a)** Spatial distribution of cellular components at the cellular level, including quiescent endothelial cells (QECs), activated endothelial cells (AECs), dead endothelial cells (DECs), monocytes (MC), dead monocytes (DMC), M1 macrophages, and M2 macrophages; **(b)** Spatial concentration field of MCP-1; **(c)** Spatial concentration field of NO; **(d)** Spatial concentration field of ROS. The spatially varying WSS serves as the prescribed external mechanical input for determining local endothelial responses; the blood-flow velocity field and flow direction are not explicitly resolved in the current model.

To further characterize the endothelial injury model, a quantitative analysis was performed. Fig 3 shows the spatiotemporal dynamics of cell phenotypes, biochemical factors, and WSS. Fig 3(a) shows the temporal dynamics of the major cell populations. At the initial state, 80 QECs were arranged along the x-axis within the simulation domain. Under lower local OSS amplitude stimulation, the number of QECs began to decrease around day 1 and remained at zero thereafter. The number of AECs increased over time, showed a slight decline between days 4 and 5, and reached a peak at days 6–7. The numbers of M1 and M2 macrophages increased with temporal fluctuations, reflecting progressive immune-cell recruitment and polarization. The numbers of DECs and DMCs also increased over time, indicating progressive endothelial injury and monocyte loss. Fig 3(b) shows the proportions of EC phenotypes. AECs accounted for the largest fraction (approximately 0.7), DECs accounted for about 0.3, and QECs accounted for nearly zero. Fig 3(c) shows the monocyte transition rates. The transition rates of pro-inflammatory M1 macrophages, anti-inflammatory M2 macrophages, and DMCs were approximately 0.5, 0.3, and 0.2, respectively, indicating a predominantly pro-inflammatory microenvironment at the early stage of injury. Fig 3(d) shows the spatial distribution of WSS. A symmetric V-shaped distribution along the x-axis was observed, with a minimum value of 5 dyn/cm^2^ at the center position (*x* = 400 µm). Fig 3(e) shows the temporal dynamics of NO and ROS concentrations. NO concentration increased rapidly at the early stage and continued to rise at the mid-to-late stages, although the rate of increase gradually slowed. In contrast, ROS concentration accumulated continuously and increased more rapidly at later stages. These results indicate progressive ROS accumulation and enhanced ROS-mediated NO consumption, which may reduce local NO bioavailability despite the continued increase in total NO concentration, thereby contributing to a progressively more oxidative microenvironment. As illustrated in Fig 3(f), the temporal profile indicates that NO generation attributed specifically to the QEC population undergoes a rapid decline toward zero around Day 1.5. It is crucial to clarify that this mathematical decline does not represent a biological cessation of basal NO synthesis within the global vascular endothelium. Rather, under the sustained, localized disturbance of low OSS at the focal lesion center, the entire regional QEC population sequentially crosses the cumulative damage threshold, systematically transitioning into the AECs. Consequently, the localized statistical contribution of QEC-derived NO is seamlessly transferred to and sustained by the compensatory, albeit impaired (0.6-fold baseline), synthesis mode of the emerging AEC population, resulting in the localized phenotype-crossover signature captured in the simulation tracking. Overall, the spatial organization and phenotypic evolution of cells reproduced the dynamic pathological processes of endothelial injury and subsequent inflammatory cell recruitment.

**Fig 3:**
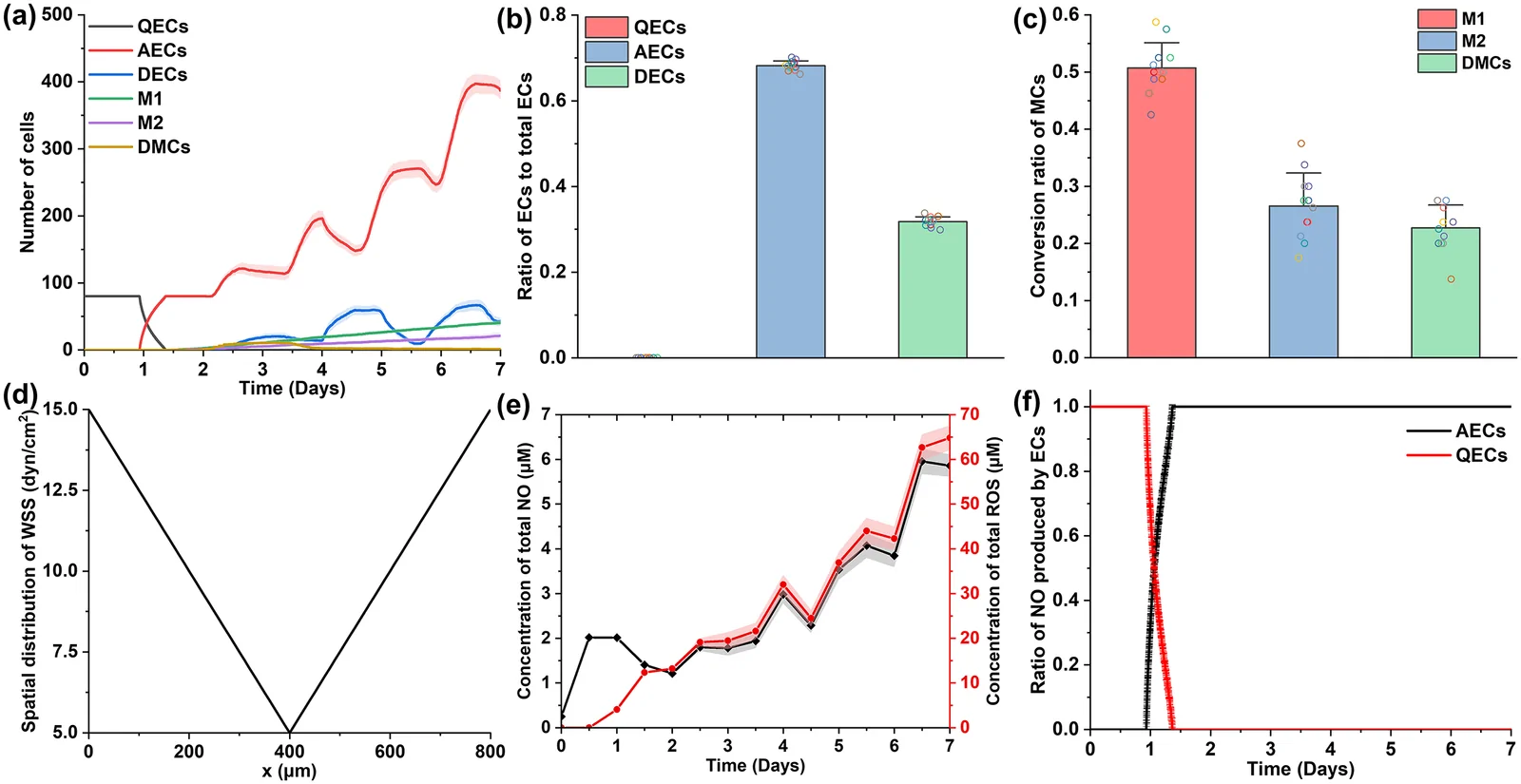
Temporal and spatial dynamics of cellular phenotypes, biochemical factors, and wall shear stress in the endothelial injury model. **(a)** Temporal changes in the number of QECs, AECs, DECs, M1 macrophages, M2 macrophages, and DMCs; **(b)** Proportion of QECs, AECs, and DECs relative to the total EC population; **(c)** Conversion ratio of M1 macrophages, M2 macrophages, and DMCs; **(d)** Spatial distribution of WSS; **(e)** Temporal changes in the concentrations of NO (black curve) and ROS (red curve); **(f)** Temporal changes in the ratio of NO produced by AECs and QECs.

### 3.2 Model validation

AKT was a key regulatory kinase in the PI3K/AKT/eNOS signaling pathway. It was regulated upstream by PI3K and influenced NO production downstream through eNOS phosphorylation (eNOSp). To investigate the effects of AKT on the delay and repair of endothelial injury, a multi-stage validation strategy was employed to verify the accuracy of the model framework (Fig 4).

**Fig 4:**
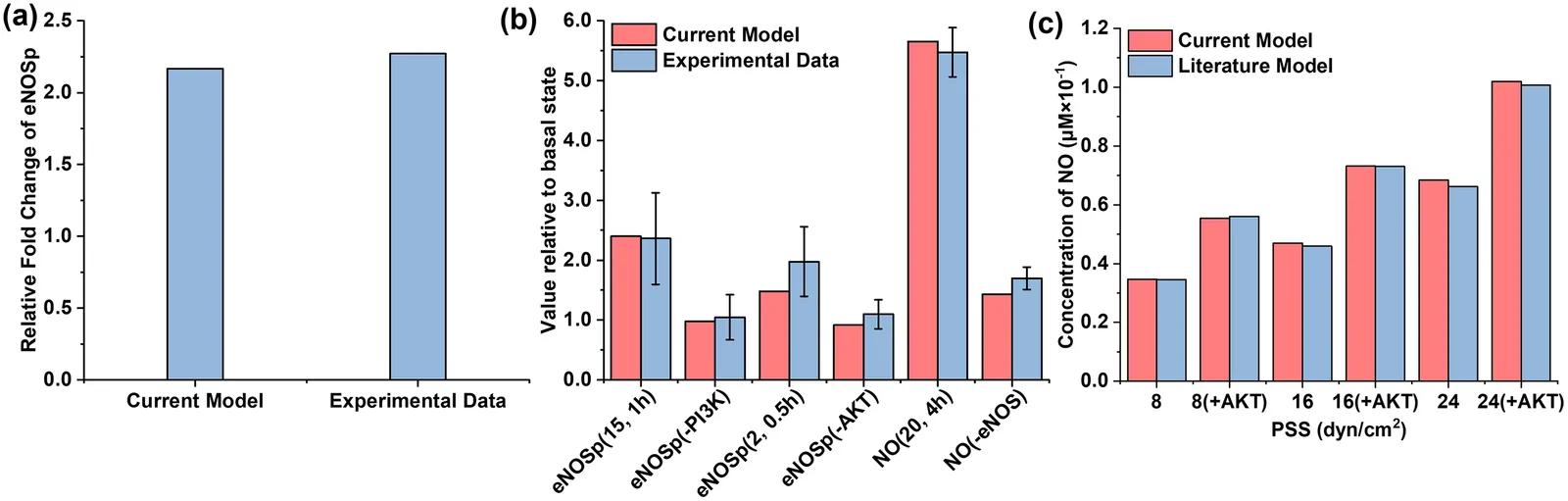
Model validation. **(a)** Comparison of the relative fold change of eNOSp between the current model simulation and literature experimental data [52] upon AKT activation/overexpression under static conditions. **(b)** Comparison between model predictions and experimental data under different PSS conditions and pathway inhibitor interventions [37,53,54]. **(c)** Comparison of NO concentrations predicted by the current model and the literature reference model under different PSS intensities (8, 16, and 24 dyn/cm^2^) with and without AKT enhancement [35].

First, to evaluate the core biochemical network independent of mechanical forces, the model’s baseline response to AKT activation under static conditions (no shear stress) was validated. As shown in Fig 4(a), a 2.5-fold increase in AKT activity in the current model predicted a 2.17-fold upregulation of eNOSp relative to the basal state. This quantitative trend aligns closely with published Western blot experimental data [52], where AKT overexpression induced a 2.27-fold increase in the eNOSp ratio, demonstrating that the baseline kinetic equations for the AKT-eNOS pathway are accurately parameterized.

Next, the model’s coupling behavior with fluid mechanics was validated under dynamic conditions. As shown in Fig 4(b), model predictions were compared with published experimental data under different pulsating shear stress (PSS) conditions and pathway inhibitor interventions. Specifically, the effects of a PI3K inhibitor on eNOS phosphorylation were examined at 15 dyn/cm^2^ PSS for 1 h [53], the effects of an AKT inhibitor on eNOS phosphorylation were examined at 2 dyn/cm^2^ PSS for 0.5 h [37], and the effects of an eNOS inhibitor on NO production were examined at 20 dyn/cm^2^ PSS for 4 h [54]. The model predictions showed consistent trends and comparable magnitudes with the experimental data, and similar responses were observed after inhibitor interventions. These results indicated that the model was able to reasonably reproduce the regulatory effects of the PI3K/AKT/eNOS pathway on eNOS phosphorylation and NO production.

Finally, Fig 4(c) further validated the model performance in predicting NO production under different PSS intensities and with or without AKT enhancement. Under PSS levels of 8, 16, and 24 dyn/cm^2^, as well as after introduction of AKT-enhanced signaling, the current model predictions closely matched those from the literature reference model [35]. These results indicated that the model reliably captured NO dynamics under varying shear stress and AKT regulatory conditions even when direct dynamic experimental data were limited.

### 3.3 Effects of OSS and AKT activation on endothelial signaling dynamics

To investigate the regulatory effects of OSS and AKT signaling activity on the endothelial signaling network, the temporal dynamics of key molecules (AKTa, eNOSp, and NO) were systematically analyzed. Fig 5a-5c showed the responses of AKTa, eNOSp, and NO under different local OSS amplitudes (5, 10, and 15 dyn/cm^2^), respectively. Under all OSS conditions, the AKTa concentration was transiently reduced at the early stage, followed by a rapid recovery and a stable plateau. Similar temporal patterns of AKTa dynamics were observed across different OSS levels (Fig 5a). The level of eNOSp remained low at early times and increased markedly at the mid to late stages. With increasing OSS, both the rise rate and the steady-state level of eNOSp were elevated (Fig 5b). The temporal profile of NO concentration was consistent with that of eNOSp (Fig 5c), but with a more gradual increase. At the mid to late stages, NO levels increased significantly with higher OSS. Fig 5d-5f illustrated the the molecular-scale responses of AKTa, eNOSp, and NO production under graded AKT activation strengths ranging from 1 to 5 times the baseline *k*_*af*5_. S2-S4 Figs showed the temporal dynamics of 16 species under OSS levels of 5, 10, and 15 dyn/cm^2^ with graded AKT activity (1–5 × baseline *k*_*af*5_). Here, a local OSS amplitude of 5 dyn/cm^2^ was used as a representative example. As shown in Fig 5(d), increasing AKT activation strength led to a clear, dose-dependent increase in AKTa concentration. At low kmakt, AKTa levels rose slowly and reached a lower steady state, whereas higher kmakt values resulted in a faster rise and a markedly elevated plateau. In Fig 5(e), eNOSp exhibited a delayed but pronounced increase following AKT activation. Both the rising rate and the steady-state level of eNOSp increased progressively with stronger AKT activation, indicating effective signal amplification from AKT to eNOSp. Similarly, NO concentration displayed a sigmoidal increase over time (Fig 5(f)). Enhanced AKT activation accelerated NO production and significantly elevated its final concentration.

**Fig 5:**
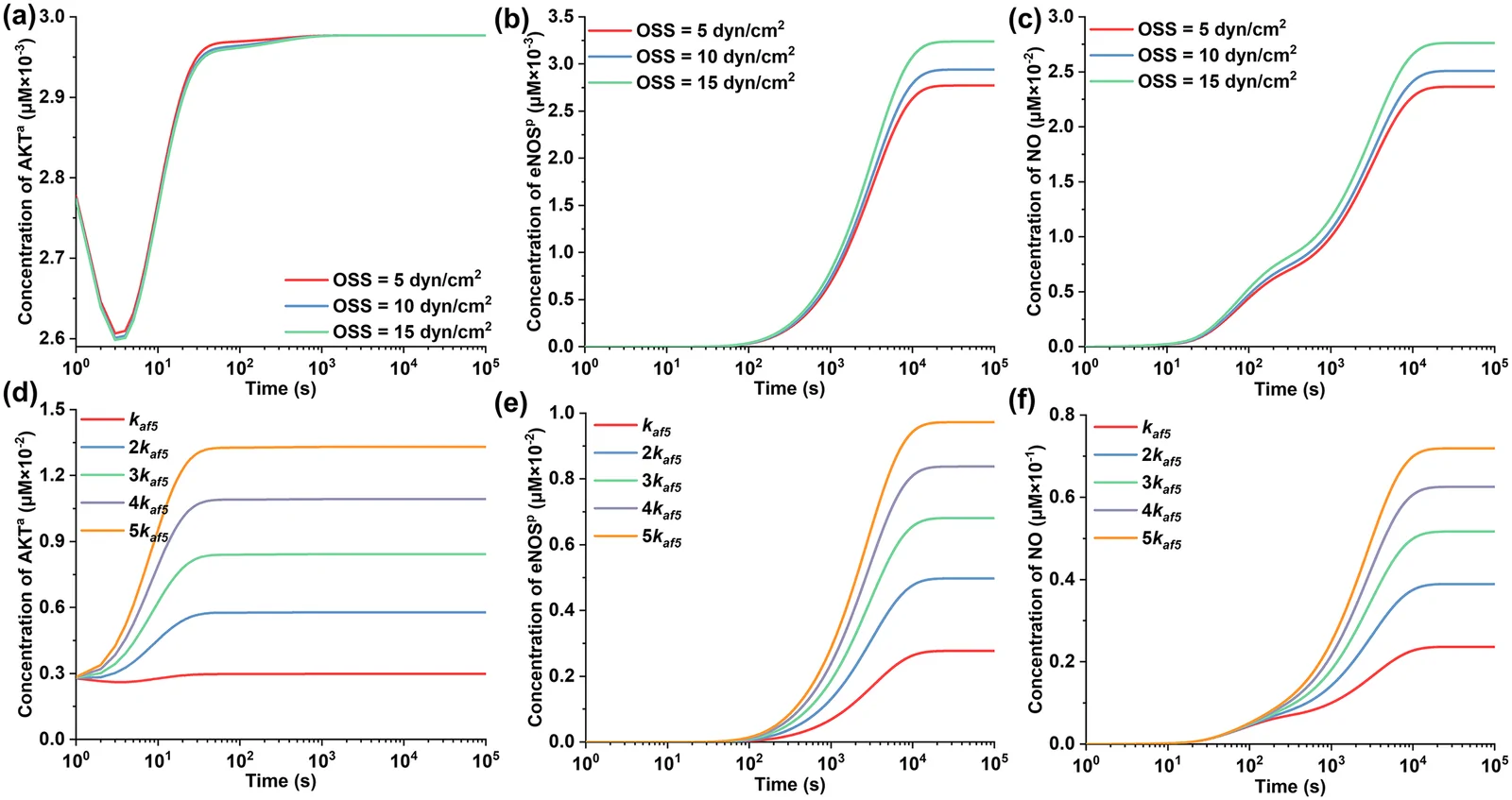
Concentration curves of AKT, eNOSp, and NO over time. Temporal evolution of AKT (a), eNOSp (b), and NO (c) concentrations under different OSS amplitudes (5, 10, and 15 dyn/cm^2^). Under OSS = 5 dyn/cm^2^, the temporal evolution of AKT **(d)**, eNOSp **(e)**, and NO (f) concentrations under different AKT activation strengths of 1-5 × baseline *k*_*af*5_.

### 3.4 Spatiotemporal regulation of endothelial injury repair by AKT treatment timing

To characterize the repair of endothelial injury induced by OSS, qualitative and quantitative analyses of endothelial repair were performed under an untreated baseline and different AKT treatment time points (Figs 6-8). Our Previous studies showed that an NO level of approximately 0.05 μM was produced in endothelial cells under stimulation with PSS = 15 dyn/cm^2^ and was associated with endothelial protection [36]. Therefore, the AKT activation strength was required to be at least 3*k*_*af*5_. In addition, ROS-mediated NO consumption was included in this study. Therefore, a fourfold AKT activation strength was selected. This parameter was used to clearly evaluate the repair effects of AKT treatment initiated at different time points after endothelial injury.

Fig 6 compared the spatiotemporal dynamics of cell phenotypes, NO, ROS, and MCP-1 among the untreated baseline (Fig 6a) and different AKT treatment initiation times, including day 1 (Fig 6b), day 3 (Fig 6c), and day 5 (Fig 6d) after injury. These results were used to evaluate the effects of treatment timing on endothelial repair. In the untreated group (Fig 6a), without any clinical intervention, the endothelial injury progressively deteriorated from days 1–7, characterized by massive ROS accumulation, severe MCP-1-mediated inflammation, and a complete failure of NO homeostasis restoration by day 7.

**Fig 6:**
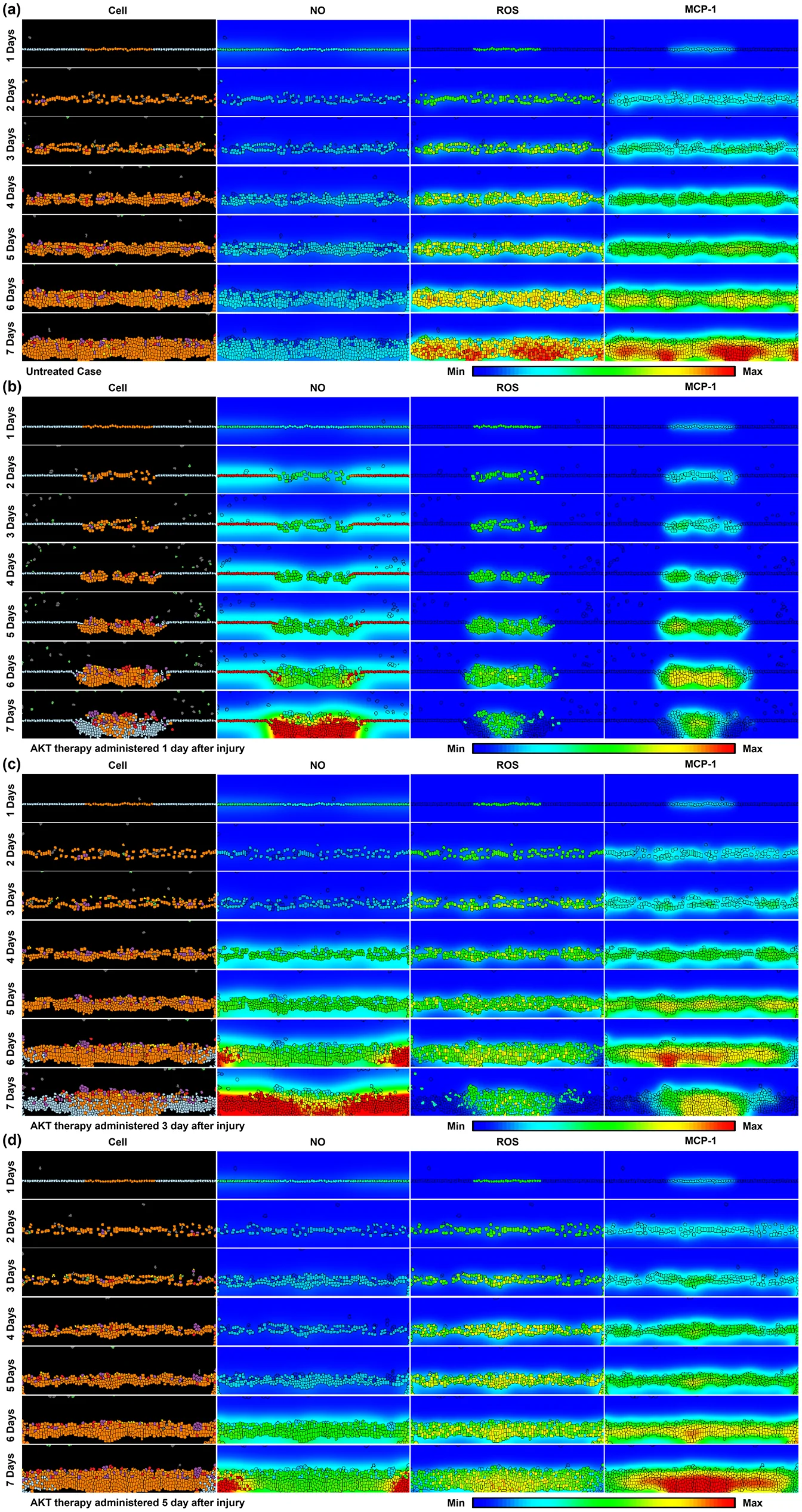
The spatiotemporal dynamics of the endothelial injury microenvironment under the untreated baseline and different AKT treatment initiation times. **(a)** Untreated case (baseline control), and AKT therapy administered at (b) day 1, (c) day 3, and (d) day 5 after injury.

When AKT treatment was initiated on day 1 after injury, in sharp contrast to the untreated group, central QECs were converted into AECs, whereas the endothelial layers on both sides remained intact (Fig 6b). By day 5, AECs on both sides of the injury center were gradually converted into QECs. MCs and DMCs were mainly distributed in the vascular lumen. The proportions of M1 and M2 macrophages within cell clusters in the injured region remained low. In addition, NO concentrations in the lateral regions were maintained within the normal range, whereas NO synthesis in the injury center was suppressed. By day 5, NO levels on both sides of the injury center were restored with the transition of AECs to QECs, which promoted repair of surrounding AECs. ROS accumulation in the repaired regions was markedly inhibited. The chemokine MCP-1 gradually increased from days 1–6 and showed a pronounced decline on day 7.

When AKT treatment was initiated on day 3 after injury, the extent of endothelial activation was increased compared with early intervention (Fig 6c). The proportion of immune cells was also increased. The overall repair effect remained significantly better than that in the untreated baseline group (Fig 6a). NO production was markedly increased only after day 5, with a delayed peak time and reduced magnitude. The inhibitory effect on ROS was weakened, and pronounced oxidative stress persisted in the injured region. MCP-1 expression remained higher than that in the early treatment group.

When AKT treatment was initiated on day 5 after injury, the extent of endothelial activation was similar to that in the untreated group (Fig 6d). The conversion of AECs to QECs was minimal. Endothelial injury progressively worsened during days 1–6. Only a small number of AECs were converted to QECs on day 7, and this was accompanied by a pronounced immune response. NO production increased markedly only on day 7. The peak level was the lowest among the three groups. ROS accumulated prominently in the injury center. Oxidative stress was not effectively controlled. MCP-1 expression was the highest among the three groups, indicating the most severe inflammatory response. Late intervention failed to effectively suppress inflammatory cell recruitment.

To further characterize cellular changes during endothelial injury repair, the temporal evolution of endothelial cell phenotypes and immune cell proportions was systematically compared under different AKT treatment initiation times (Fig 7). Fig 7(a) showed the temporal evolution of the QECs proportion. When AKT treatment was initiated 1 day after injury, the QECs proportion rapidly decreased on day 1, followed by a distinct step-like decline from day 1 to day 5. This step-like trajectory resulted from the cumulative damage-threshold rule implemented in the model. QECs were converted to AECs when the accumulated damage exceeded the predefined threshold. The QECs proportion subsequently began to recover on day 6. In contrast, for the 3-day and 5-day delayed treatment groups, the QECs proportion plummeted to zero around day 1.2. The 3-day group began to recover on day 5.5, whereas the 5-day group showed a delayed and weaker recovery starting around day 6.5, resulting in the lowest final proportion by day 7.

**Fig 7:**
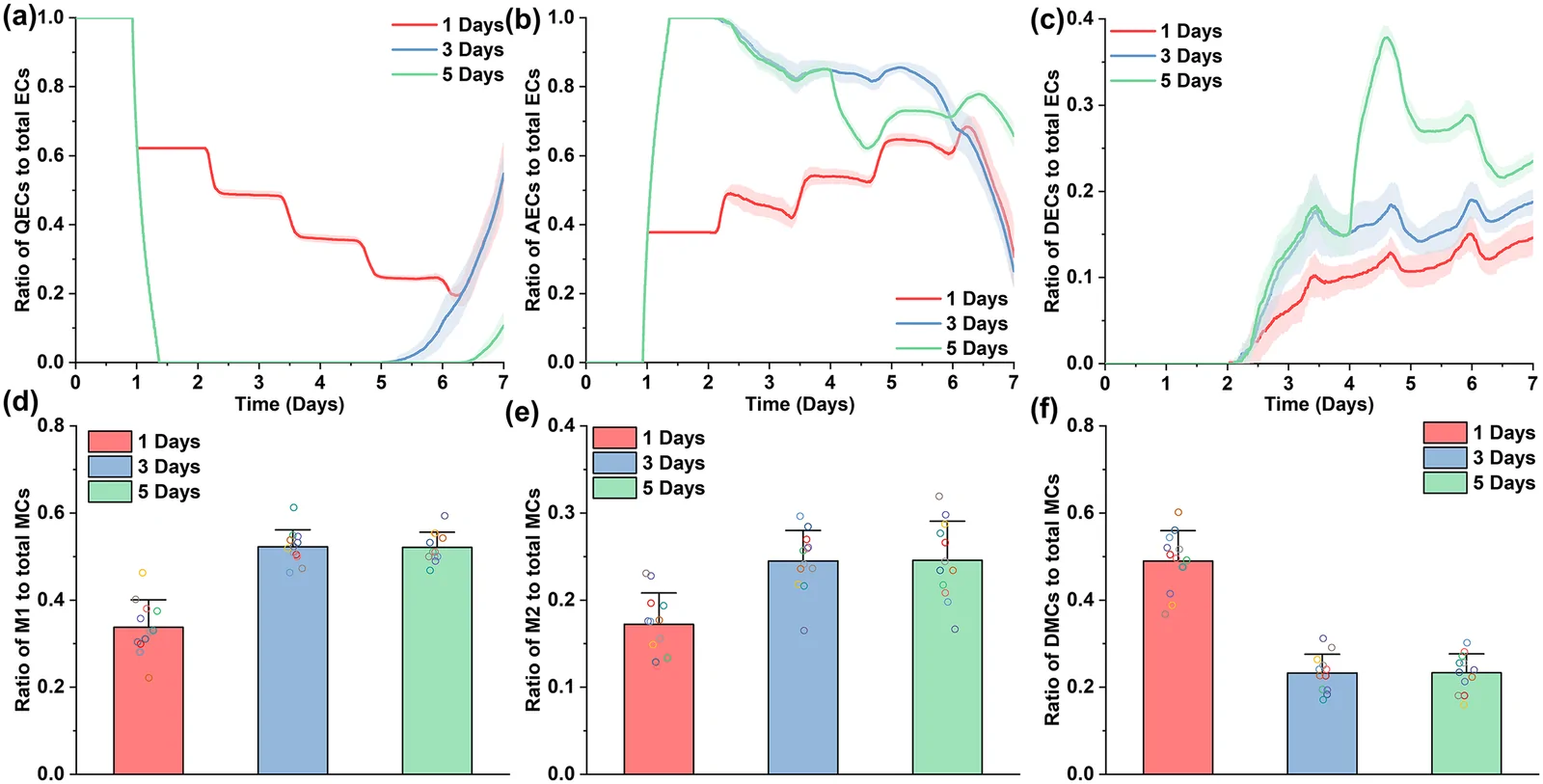
Dynamic changes in endothelial cell phenotypes and immune cell proportions under different AKT treatment initiation times. Time-course profiles of the proportions of **(a)** QECs, **(b)** AECs, and **(c)** DECs relative to total endothelial cells; Proportions of **(d)** M1, **(e)** M2, and **(f)** DMCs relative to total monocytes at day 7.

Fig 7(b) illustrates the temporal changes in the AECs proportion. In all three groups, the AECs proportion increased rapidly at early stages, maintaining a plateau near 1.0 until day 2. Following AKT intervention on day 1, the AEC proportion showed a transient decrease, followed by step-like changes during days 2–5. This step-like pattern corresponds to the sudden phenotypic transition of cells that have sequentially reached their switching thresholds under continuous AKT activation. It then showed a marked decline after day 6, signaling a reverse phenotypic transition from AECs back to QECs as the system successfully accumulated a sufficient health/recovery threshold. In contrast, delaying treatment to day 3 or day 5 sustained higher AEC levels for a longer duration. Notably, when treatment was initiated on day 3, the AECs proportion changed more gradually and began to decline after day 5. For the 5-day treatment group, the AECs proportion remained identical to the 3-day group initially, sharing a system-inherent fluctuation with a sharp drop on day 4.5, and diverged only after the AKT activation was triggered on day 5, followed by a second decline around day 6.5.

Fig 7(c) shows the temporal evolution of the DECs proportion. In all groups, the DECs proportion increased over time, but the magnitude of the increase depended on the timing of AKT treatment. When treatment was initiated 1 day after injury, the DECs proportion remained the lowest. When treatment was initiated 3 days after injury, the DECs proportion remained at an intermediate level. When treatment was initiated 5 days after injury, the DECs proportion was the highest and showed a pronounced peak around day 4.5. These results showed that delayed AKT treatment was associated with a higher DEC proportion and poorer endothelial recovery in the simulations.

Fig 7d-7f show the proportions of M1 macrophages (d), M2 macrophages (e), and DMCs (f) relative to total monocytes at day 7 (their comprehensive temporal dynamics are shown in S5 Fig). When AKT treatment was initiated 1 day after injury, the proportions of M1 and M2 on day 7 were lower than those in the 3-day and 5-day treatment groups. Similar proportions were observed between the 3-day and 5-day treatment groups (approximately 0.52 for M1 and 0.25 for M2). In contrast, the proportion of DMCs was significantly higher in the 1-day treatment group. Quantitatively, when treatment was initiated on day 1, the final proportion of DMCs was approximately 0.49, whereas M1 and M2 reached approximately 0.34 and 0.17, respectively. When treatment was initiated on day 3 or day 5, the final proportion of M1 was approximately 0.52, and similar proportions of M2 and DMCs were observed, both at approximately 0.24.

To further clarify endothelial phenotypic transitions, the production of NO and ROS and the contributions from different endothelial subpopulations were quantitatively analyzed under different AKT treatment initiation times. The temporal evolution of these parameters was systematically compared (Fig 8). Fig 8(a) showed the temporal evolution of total NO production by all endothelial cells. In all groups, total NO increased over time. The growth pattern was dependent on the timing of AKT treatment. When treatment was initiated 1 day after injury, total NO remained lower than in the day 3 and day 5 groups, but the increase occurred earliest and was observed on day 1. When treatment was initiated 3 days after injury, NO accumulated most rapidly and reached the highest peak, with a marked increase starting on day 3. When treatment was initiated 5 days after injury, NO levels were intermediate, but the increase was delayed. Fig 8(b) showed the temporal evolution of total ROS produced by AECs. When AKT treatment was initiated 1 day after injury, ROS levels produced remained the lowest and increased slowly, and the absolute values were still markedly lower than those in the other groups even at the peak stage. When treatment was initiated 3 days after injury, ROS accumulation increased markedly during the middle to late stages and reached a peak around days 6–7, followed by a slight decline. In contrast, when treatment was initiated 5 days after injury, early ROS accumulation was similar to that in the day 3 group and slightly lower during days 4–6, but it increased rapidly at later stages and reached the highest peak. Fig 8(c) showed the temporal changes in the relative contribution of QECs to NO production. When AKT treatment was initiated 1 day after injury, the contribution of QECs to NO production gradually decreased and reached a minimum of approximately 0.4 on day 6, then rapidly increased and rose to about 0.8. In contrast, when treatment was initiated 3 or 5 days after injury, QECs contributed little to NO production during the early phase and recovered only at later stages of repair. In the day 3 treatment group, QEC contribution began to recover on day 5 and reached a level similar to that of the early treatment group at later times. In the day 5 treatment group, the recovery was further delayed and the final contribution remained the lowest. Fig 8(d) showed the relative contribution of AECs to NO production. An opposite temporal trend to that of QEC-derived NO contribution was observed.

**Fig 8:**
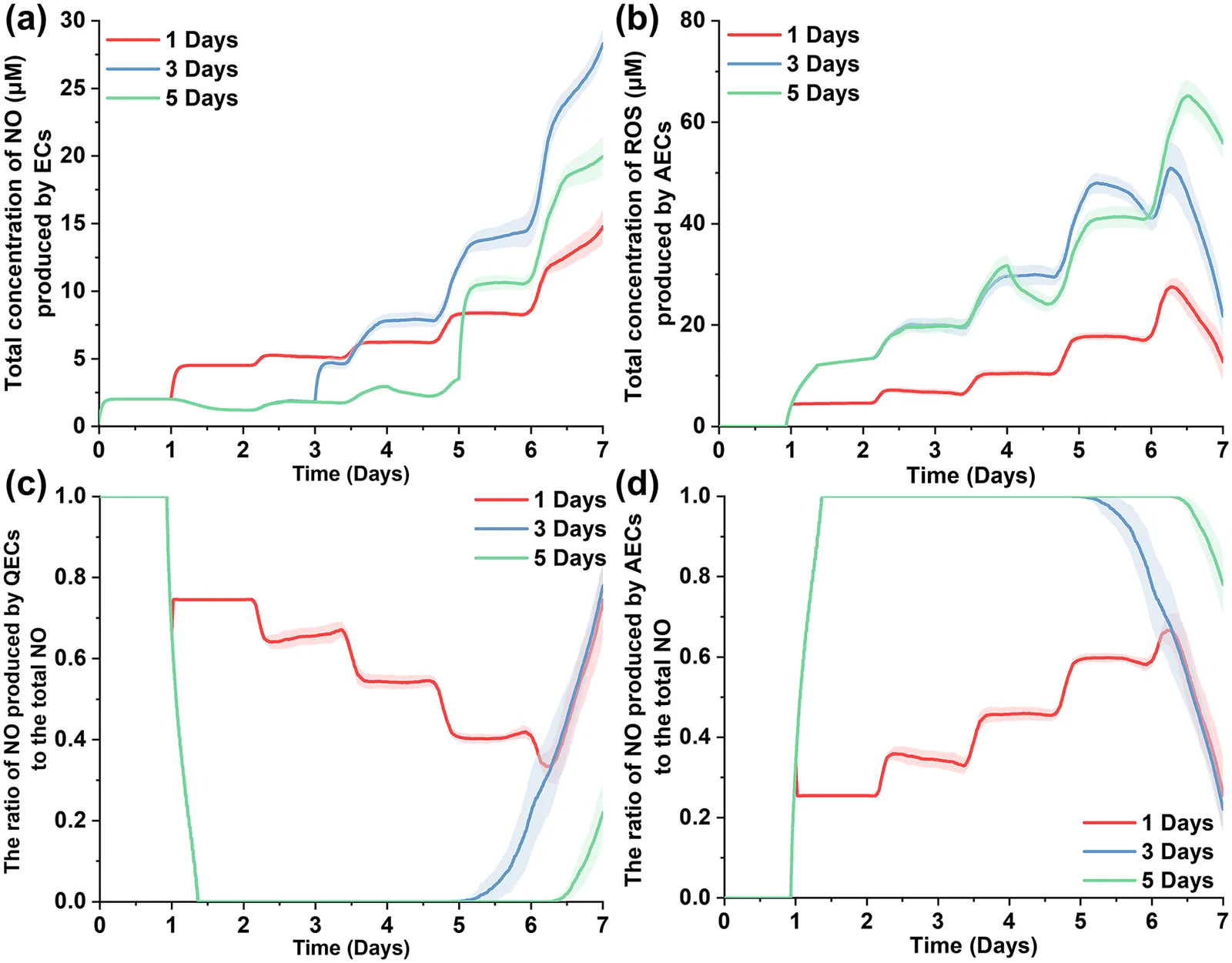
Temporal dynamics of NO production, ROS generation, and cellular source contributions under different AKT treatment initiation times. **(a)** Temporal evolution of total NO production by all endothelial cells. **(b)** Temporal evolution of total ROS produced by AECs. **(c)** Temporal changes in the proportion of total NO contributed by QECs. **(d)** Temporal changes in the proportion of total NO contributed by AECs.

### 3.5 Spatiotemporal regulation of endothelial injury repair by different AKT activation strengths

To clarify the spatiotemporal regulation of endothelial injury repair by different AKT activation strengths, the intervention was initiated on day 3 after injury. This time point represented a critical transition from inflammation to repair. It allowed clearer differentiation of repair dynamics under different AKT activation strengths. Therefore, the spatial distribution of ECs and the spatiotemporal dynamics of NO, ROS, and MCP-1 were analyzed at AKT activation strengths: 3.4 *k*_*af*5_ (weak activation), 3.8 *k*_*af*5_ (moderate activation), and 4.2 *k*_*af*5_ (strong activation) (Fig 9). Under weak AKT activation (3.4 *k*_*af*5_, Fig 9a), the first transition from AECs to QECs was delayed, and repair was initiated late (146 h). NO levels remained low and increased slowly, preventing the formation of a stable high-NO region. Meanwhile, ROS and MCP-1 levels remained elevated in the injured area, and inflammation resolved slowly. These results indicated that weak AKT activation was insufficient to drive endothelial phenotype transition and functional repair. Under moderate AKT activation (3.8 *k*_*af*5_, Fig 9b), the transition from AECs to QECs was observed earlier (134 h) and progressively expanded (168 h). NO levels were markedly increased and became more uniform in the injured region, indicating improved endothelial functional recovery. ROS and MCP-1 levels decreased significantly over time, and the inflammatory microenvironment was effectively alleviated. At this activation strength, endothelial repair, NO production, and inflammation control exhibited a favorable dynamic balance. Under strong AKT activation (4.2 *k*_*af*5_, Fig 9c), the transition from AECs to QECs was observed at the earliest time point (124 h), and repair was initiated most rapidly. NO levels increased rapidly at early stages and formed high-concentration regions. However, ROS levels were markedly reduced at later stages, and MCP-1 levels were suppressed to a minimum level compared to the weak and moderate AKT activation groups. Among the three tested activation strengths, 3.8 *k*_af5_ showed the most balanced combination of earlier QEC recovery, increased NO, and reduced ROS and MCP-1. In comparison, strong AKT activation (4.2 *k*_af5_) accelerated the AEC-to-QEC transition and produced the greatest reduction in ROS and MCP-1 among the tested conditions.

**Fig 9:**
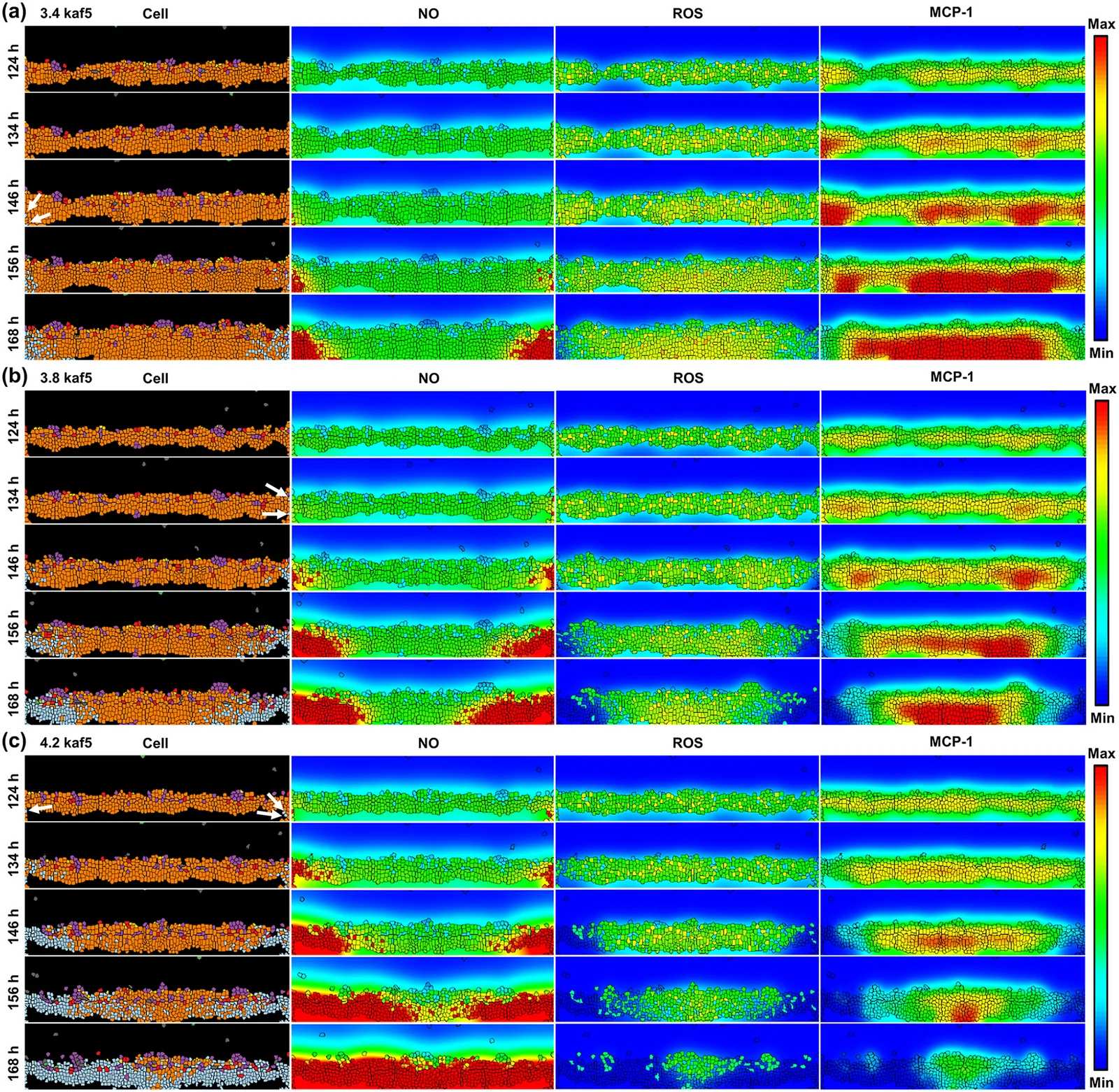
Spatiotemporal evolution of the endothelial injury microenvironment under different AKT activation strengths with treatment initiated 3 days after injury. AKT activation strengths were set to 3.4 *k*_*af*5_ **(a)**, 3.8 *k*_*af*5_ **(b)**, and 4.2 *k*_*af*5_ **(c)**, respectively. The arrows indicated the time point at which the transition from AECs to QECs was first observed after injury.

To clarify the regulatory effects of different AKT activation strengths on endothelial functional states and the redox microenvironment, quantitative analyses were performed on endothelial phenotype distributions and the temporal dynamics of NO and ROS (Fig 10). With increasing AKT activation strength, the transition of AECs to QECs occurred at earlier time points. The transition was observed at approximately 6, 5.5, and 5 days under **3.4, 3.8, and 4.2 *k***_**af5**_, respectively. Accordingly, QEC recovery occurred earlier with stronger AKT activation (Fig 10a). The AEC proportion decreased progressively during the repair phase, showing a trend opposite to that of QECs. Under 4.2 ***k***_**af5**_, the AEC proportion approached zero at the end of the simulation (Fig 10b). In contrast, the DEC proportion showed no substantial differences among the three AKT activation strengths (Fig 10c).

**Fig 10:**
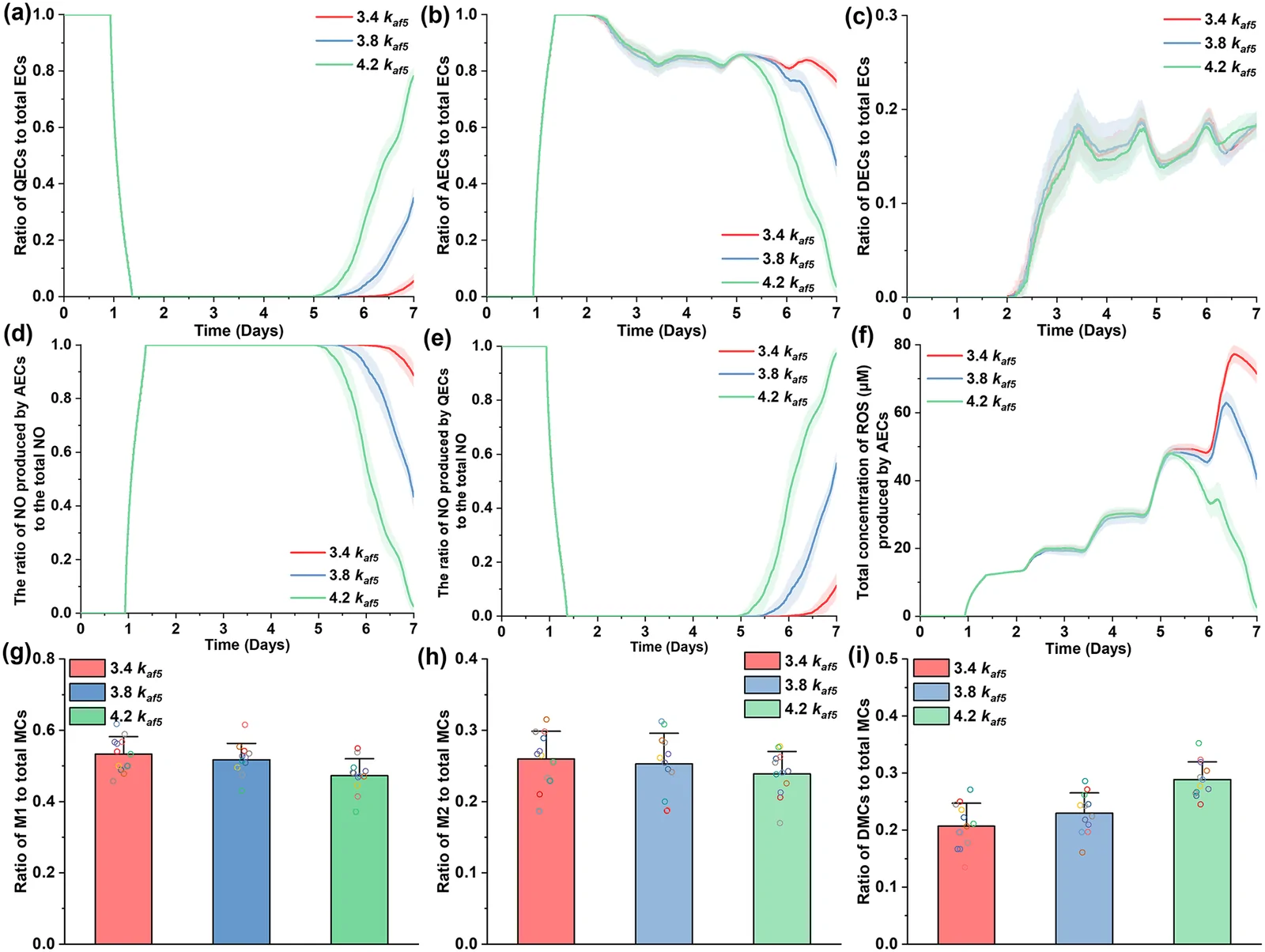
Temporal evolution and endpoint comparison of endothelial cell phenotypes, mediator production, and monocyte phenotypic transitions under different AKT activation strengths. Time courses of the ratios of **(a)** QECs, **(b)** AECs, and **(c)** DECs to the total EC population; Time-dependent contributions of **(d)** AECs and **(e)**QECs to NO production; **(f)** Temporal changes in total ROS production by AECs; Capacities of MCs to differentiate into the **(g)** M1 phenotype, the **(h)** M2 phenotype, and **(i)** DMCs.

The relative contribution of AECs to NO production began to decrease at approximately day 6.5, day 5.5, and day 5 under 3.4, 3.8, and 4.2 ***k***_**af5**_, respectively(Fig 10d). Correspondingly, the contribution of QECs to NO production increased (Fig 10e). Under 4.2 ***k***_**af5**_, NO production was predominantly contributed by QECs at the later stage. ROS production by AECs initially increased and subsequently decreased. The ROS peak occurred at approximately day 6.6, day 6.5, and day 5.2 under 3.4, 3.8, and 4.2 ***k***_**af5**_, respectively (Fig 10f). The decline in ROS was most pronounced under strong AKT activation.

Fig 10g-10i showed that the proportions of M1 and M2 macrophages were only modestly affected by different AKT activation strengths, whereas the proportion of DMCs slightly increased with increasing activation strength. In addition, the total proportion of immune cells gradually decreased as the AKT activation strength was reduced. At 4.2 ***k***_**af5,**_ the total proportion of immune cells was 0.79, with proportions of 0.53 for M1 and 0.26 for M2. At 3.8 ***k***_**af5**_, the total proportion was 0.77, with proportions of 0.52 for M1 and 0.25 for M2. At 3.4 ***k***_**af5**_, the total proportion decreased to 0.70, with proportions of 0.45 for M1 and 0.25 for M2. Furthermore, S6 Fig presented the time-dependent dynamics of MC differentiation into M1 macrophages, M2 macrophages, and DMCs under different AKT activation strengths.

## 4. Discussion

Based on our previous work, a multiscale computational model was constructed in this study [36]. This model was designed to clarify the regulatory mechanisms of the AKT signaling pathway in endothelial injury and repair induced by OSS. Many models have investigated the pathological progression of endothelial injury and the effects of drug interventions [28–32,55]. However, the dynamic balance between injury and repair under shear stress has rarely been explored through AKT signaling at a spatiotemporal scale. As shown in Figs 2 and 3, the spatial heterogeneity of cellular components and biochemical factors was successfully simulated. The dynamic evolution of endothelial phenotypes, inflammatory cell recruitment, and polarization was also captured over time. In addition, model predictions of eNOSp and NO production were compared with experimental data under various conditions [35,37,53,54] (Fig 4). The reliability of this multiscale model was validated at both quantitative and qualitative levels. These results indicate that OSS-induced endothelial dysfunction is effectively characterized by the model. Furthermore, a robust theoretical foundation is provided for promoting endothelial repair by modulating AKT to enhance eNOS activity.

The spatial heterogeneity of WSS is a key factor in determining the spatially differential response of ECs. In this study, a V-shaped symmetric distribution was established to simulate the complex hemodynamic environment of susceptible sites, such as coronary artery bifurcations or curvatures (Fig 3d). In these regions, low OSS generated by flow separation and recirculation is the dominant factor inducing endothelial injury [56]. Significant endothelial activation was observed in regions exposed to low OSS, while homeostasis was better maintained under physiological shear stress (Fig 11a). In the central region (low OSS zone), a large-scale transition from QECs to AECs occurred (Fig 3b). This transition was accompanied by high expression of the pro-inflammatory factor MCP-1 (Fig 2b). A chemotactic axis was formed by the MCP-1 concentration gradient, which drove the directional recruitment of monocytes and their polarization into M1 macrophages (Figs 2a, 3c). Furthermore, intense oxidative stress was observed in the injured regions. High concentrations of ROS reacted with NO, which was already limited in synthesis, leading to a significant decrease in local NO bioavailability (Fig 2c-2d). This biochemical environment of “high ROS and low NO” characterizes the dysfunctional state of ECs under low OSS stimulation [9]. Although higher eNOS/NO levels were maintained in the lateral regions due to relatively higher OSS intensity (Fig 5b-5c), the entire domain remained under the stress of OSS-induced injury. Clinical and computational studies have confirmed that this OSS intensity is closely related to the anatomical localization of coronary plaques [57,58]. Additionally, it was revealed that eNOS phosphorylation levels could be significantly increased by enhancing AKT activity [35,59]. This mechanism effectively compensated for the NO deficiency induced by OSS at the molecular level (Fig 5d-5f). These findings validate the dominant role of biomechanical-biochemical coupling in endothelial injury. A solid theoretical foundation is thus provided for the precision repair of the endothelium by modulating the AKT pathway.

**Fig 11:**
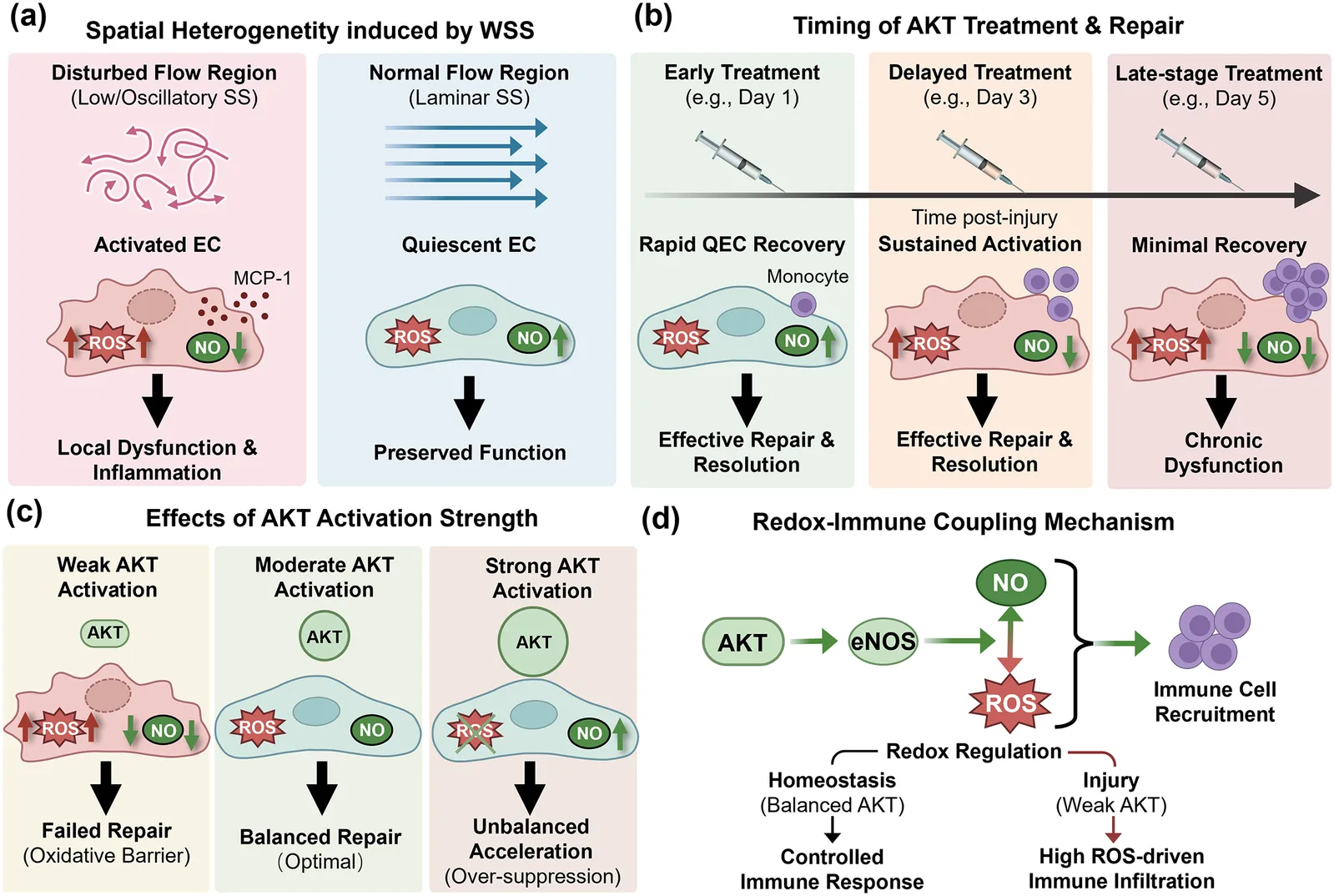
Multifactorial regulation of endothelial injury and repair outcomes. **(a)** Spatial heterogeneity in endothelial function induced by WSS. **(b)** Timing of AKT treatment post-injury and its impact on repair outcomes. **(c)** Effects of AKT activation strength on repair success. **(d)** Redox-immune coupling mechanism linking AKT activity to homeostasis or chronic dysfunction.

A key finding of this study is that the efficacy of endothelial repair is highly dependent on the timing of AKT activation (Fig 11b). Early intervention at 1 day post-injury most effectively maintained endothelial integrity (Fig 6a), delayed the progression of endothelial injury (Fig 7b), and accelerated the recovery of the QEC population (Fig 7a). Although the peak total NO production in the Day 1 group was not the highest among all groups (Fig 8a), NO increased rapidly after intervention, while ROS remained at the lowest level (Fig 8b). Mechanistically, early AKT activation enhances eNOS-mediated NO production before extensive oxidative stress is established. Increased NO promotes endothelial functional recovery and facilitates the transition from AECs to QECs. As the AEC population decreases, cellular ROS production is reduced, which further limits ROS-mediated NO consumption and favors restoration of redox homeostasis. In contrast, delayed AKT activation occurs after substantial ROS accumulation, resulting in greater consumption of newly generated NO and therefore lower effective NO bioavailability.

The phenotype-specific contribution to NO production further supports this timing-dependent mechanism (Fig 8c, 8d). In the Day 1 group, the proportion of NO contributed by QECs reached a minimum of approximately 0.4 on day 6 before rapidly recovering to 0.8 (Fig 8c). This shift indicates a successful transition of endothelial function from a compensatory state (driven by AECs) back to a physiological steady state (driven by QECs). In contrast, a pronounced delay in repair was observed in the Day 5 group, where the contribution of QECs recovered slowly and reached the lowest final value (Fig 8c). Due to this lack of repair momentum, the injured region was exposed to high oxidative stress for an extended duration, which is evidenced by the late-stage ROS burst observed in the Day 5 group (Fig 8b). Consequently, the imbalance of redox homeostasis appears to be an important mechanism underlying the reduced efficacy of delayed treatment. Although the highest total NO production was achieved by the Day 3 group in the later stages (Fig 8a), the repair quality remained inferior to the Day 1 group. This disparity is explained by the reduction in NO bioavailability. In the delayed treatment groups, the proportion of AECs remained high (Fig 7b), resulting in excessive ROS production. The generated NO was rapidly neutralized by ROS and converted into toxic ONOO-. This observation aligns with the findings of Wang et al. regarding eNOS-like biomimetic nanomaterials, which demonstrate that providing NO without simultaneously inhibiting oxidative stress is insufficient for effective tissue repair [60].

Beyond treatment timing, AKT activation strength was also shown to critically regulate endothelial repair dynamics (Fig 11c). The response was non-linear rather than increasing proportionally with AKT activation. Under weak AKT activation (3.4 *k*_*af*5_), NO restoration was insufficient to overcome the oxidative and inflammatory microenvironment, resulting in delayed AEC-to-QEC transition, persistently elevated ROS and MCP-1, and slower QEC recovery (Figs 9a and 10a). In contrast, moderate AKT activation (3.8 *k*_*af*5_) induced an earlier AEC-to-QEC transition and established a more uniform high-NO environment, while progressively reducing ROS and MCP-1 (Fig 9b). This non-linear response can therefore be explained by the balance between restoration of NO signaling and normalization of the redox microenvironment. Rather than AKT activation alone, sufficient NO restoration together with progressive control of oxidative stress appears to determine the quality of endothelial repair.

Strong AKT activation (4.2 *k*_*af*5_) produced the earliest AEC-to-QEC transition and the most rapid reduction in ROS (Figs 9c and 10f). However, MCP-1 and other inflammatory signals were also more strongly suppressed. Although the present model does not explicitly simulate all downstream consequences of strong AKT activation, these results suggest that maximal pathway activation may alter the balance between endothelial recovery, redox signaling, and inflammatory regulation. Thus, the simulation suggests an activation-strength-dependent response within the tested parameter range, in which sufficient AKT activation promotes repair, whereas stronger activation does not necessarily produce the most balanced microenvironmental response. This interpretation is consistent with our previous work showing that moderate hemodynamic intervention is more favorable for endothelial function than excessively high or low stimulation [61]. Therefore, precise regulation of AKT activity, rather than maximal activation, may be important for maintaining vascular homeostasis.

A further finding of this study is the coupling between redox regulation and immune dynamics during endothelial repair (Fig 11d). AKT activation not only promotes restoration of endothelial function but also indirectly influences the inflammatory microenvironment. Restoration of endothelial NO production reduces oxidative stress and promotes the transition of AECs toward the quiescent phenotype. The resulting decrease in AECs reduces cellular sources of both ROS and MCP-1. Lower MCP-1 levels weaken the chemotactic signal for continued monocyte recruitment, while reduced ROS limits further endothelial dysfunction. Because the M1:M2 differentiation ratio was predefined as 2:1 in the current model, the simulations were not designed to independently evaluate AKT-dependent changes in macrophage polarization preference. Nevertheless, AKT activation can indirectly influence the abundance of M1 and M2 macrophages by modifying the endothelial redox and chemotactic microenvironment. Reduced MCP-1 levels weaken monocyte recruitment, thereby affecting the numbers of downstream macrophage populations. Thus, the immune effects captured by the present model mainly reflect AKT-dependent regulation of monocyte recruitment rather than direct regulation of the M1:M2 polarization ratio. Instead, its effects on immune dynamics are mainly mediated through restoration of endothelial redox homeostasis and modification of the local chemotactic environment.

The timing of this redox–immune regulation is also important. Early intervention rapidly reduced MCP-1 expression and limited the establishment of a sustained inflammatory microenvironment. Spatially, QECs at the margins of the injured region formed a repair front that progressively advanced toward the center (Fig 6). In contrast, delayed intervention allowed prolonged accumulation of ROS and inflammatory mediators in the central injured region, which was associated with greater endothelial damage and delayed repair. Taken together, these results indicate that successful endothelial repair depends not only on activation of the AKT/eNOS pathway but also on timely restoration of the coupled NO-ROS and endothelial-immune microenvironment.

Below are 5 innovation-focused major findings:

1. A spatially resolved multiscale model was developed that quantitatively links heterogeneous wall shear stress, endothelial phenotypic transitions, immune cell dynamics, and AKT/eNOS/NO/ROS signaling during endothelial injury and repair.
2. The model revealed that endothelial dysfunction is driven by spatial mismatch between NO production and ROS consumption, rather than by global NO deficiency, explaining localized injury under disturbed flow.
3. A critical therapeutic time window for AKT activation was identified, in which early intervention effectively restored quiescent endothelial cells, suppressed oxidative stress, and limited inflammatory cell recruitment.
4. Endothelial repair exhibited a non-linear dependence on AKT activation strength, with moderate activation achieving optimal balance between NO restoration, ROS control, and inflammatory resolution.
5. Within the current model framework, AKT-mediated changes in the inflammatory microenvironment were primarily associated with redox normalization and altered monocyte recruitment, whereas direct AKT-dependent changes in the M1:M2 polarization ratio were not independently evaluated.

Several limitations must be considered despite the significant insights provided by this multiscale model regarding AKT-mediated endothelial repair. First, the biochemical signaling network is primarily focused on the AKT/eNOS/NO/ROS axis. The complex crosstalk involving other pathways, such as Notch and TGF-β, is simplified in the current study although they contribute to endothelial phenotypic transitions. Furthermore, while the non-linear Michaelis-Menten formulation effectively captures the reciprocal chemical neutralization between NO and ROS molecules, the downstream downstream biological effects of their reaction product, peroxynitrite (ONOO-), were neglected. In *vivo*, ONOO- acts as a potent oxidant that can trigger eNOS uncoupling, establishing a secondary positive feedback loop of oxidative stress. Incorporating the explicit kinetic feedback of ONOO- and eNOS uncoupling represents a crucial direction for future model extensions. Second, the lateral progression of repair fronts is effectively captured by the current 2D spatial framework. However, the 3D structural complexity of the vascular wall is neglected, and the influence of the internal elastic lamina on molecular diffusion is not accounted for. Regarding the immune microenvironment, monocyte-to-macrophage transitions are prioritized by the model. Notably, monocyte recruitment was simplified as a function regulated by AEC density, without explicitly integrating the direct impact of hemodynamics on monocyte adhesion dynamics, such as integrin-mediated adhesion probability variations under altered blood flow. The roles of other immune cells, such as neutrophils, and a broader spectrum of cytokines remain to be integrated in future work.

## 5. Conclusion

In this study, a multiscale model was developed to quantitatively link heterogeneous WSS, endothelial phenotypic transitions, immune cell dynamics, and AKT/eNOS/NO/ROS signaling. It was revealed that endothelial dysfunction is driven by the spatial mismatch between NO production and ROS consumption rather than global NO deficiency. This mechanism successfully explains the localization of endothelial injury under disturbed flow conditions. Furthermore, a critical therapeutic time window for AKT activation was identified. It was demonstrated that early intervention most effectively restores QECs, suppresses oxidative stress, and limits inflammatory cell recruitment. The repair process exhibited a non-linear dependence on AKT activation strength, where an optimal balance between NO restoration and inflammatory resolution was achieved through moderate activation. Finally, it was observed that the AKT-mediated attenuation of inflammation is primarily achieved through the redox normalization of the endothelial microenvironment rather than direct modulation of macrophage polarization. These findings provide a theoretical foundation for the precision treatment of vascular injuries and emphasize the importance of timing and dosage in AKT-targeted therapies.

## Supporting information

S1 Fig Comparison between the PALOS and its numerical counterpart, confirming consistency and accuracy. (a) Time-course profiles of the 16 molecular species obtained using the numerical solver and the PALOS analytical solution. (b) Error analysis between the PALOS solution and the numerical results. (c) Computational time required for the multiscale model when solved numerically versus using the PALOS analytical approach.
(TIF)

S2 Fig Temporal dynamics of the signaling network species under OSS = 5 dyn/cm^2^ with graded AKT activation strengths (1× to 5 × baseline). (TIF)

S3 Fig Temporal dynamics of the signaling network species under OSS = 10 dyn/cm^2^ with graded AKT activation strengths (1× to 5 × baseline). (TIF)

S4 Fig Temporal dynamics of the signaling network species under OSS = 15 dyn/cm^2^ with graded AKT activation strengths (1× to 5 × baseline). (TIF)

S5 Fig Temporal dynamics of monocyte-derived cell proportions under different AKT treatment initiation times. Time-course profiles illustrate the ratios of (a) M1 macrophages, (b) M2 macrophages, and (c) dead monocytes (DMCs) relative to the total monocyte (MC) population.
(TIF)

S6 Fig Temporal dynamics of monocyte phenotypic transitions under different AKT activation strengths. Time-course profiles represent the ratios of (a) M1 macrophages, (b) M2 macrophages, and (c) dead monocytes (DMCs) relative to the total monocyte (MC) population.
(TIF)

S1 Table Model parameters of the WSS-NO pathway. (XLSX)

S2 Table Initial values of all non-zero components in the WSS-NO pathway. (XLSX)

S1 Text The ODE model of the WSS-NO pathway. (DOCX)
