# Supplementary material for: Multiscale modeling reveals non-linear dynamics of AKT-mediated NO-ROS crosstalk in shear stress-induced endothelial phenotype
Source: PLoS Comput Biol. 2026 Oct 21;22(10):e1014860. doi: 10.1371/journal.pcbi.1014860 (PMC13705609; doi:10.1371/journal.pcbi.1014860)
Supplement: Supplementary file 1 [file pcbi.1014860.s009.docx]

**Multiscale modeling reveals non-linear dynamics of AKT-mediated NO-ROS crosstalk in shear stress-induced endothelial phenotype**

Yu-yuan Zhang ^1,2^, Yi-Teng Wang ^1,2^, Yan Qiu ^1,2^, Yong-Jiang Li ^1,2,^*, Dong Chen ^1,^*, Kai-Rong Qin ^1,2,^*

1. Institute of Cardio-Cerebrovascular Medicine, Central Hospital of Dalian University of Technology, Dalian, Liaoning, P.R. China
2. School of Biomedical Engineering, Faculty of Medicine, Dalian University of Technology, Dalian, Liaoning, P.R. China

*****yongjiangli@dlut.edu.cn (Yong-Jiang Li); neuro-chen@dlut.edu.cn (Dong Chen) and krqin@dlut.edu.cn (Kai-Rong Qin)

**The ODE model of the WSS-NO pathway**

Wall shear stress (WSS) stimulates endothelial nitric oxide (NO) production through integrins, G-protein coupled receptors, ion channels, and related mechanotransduction pathways. This process is described by a system of 16 ordinary differential equations (Eqs. S1-S16). The corresponding parameters are listed in Table S1 (Model parameters of the WSS-NO pathway), and the initial values of all non-zero variables are provided in Table S2.

$\frac{\text{d[G}^{\text{a}}\text{]}}{\text{d}t}=k_{af1}\tanh(\frac{\pi\tau_{\text{pss}}\text{(}x,y,t\text{)}}{\Lambda}+1)([\text{G}]_{\text{tot}}-\text{[G}^{\text{a}}\text{]})-k_{ar1}[\text{G}^{\text{a}}]$ (S1)

$\frac{\text{d[IP}_{3}\text{]}}{\text{d}t}\text{ = }k_{1}\text{[G}^{\text{a}}\text{][PIP}_{2}\text{]}-k_{d1}\text{[IP}_{3}\text{]}$ (S2)

$\begin{matrix} \frac{\text{d[PIP}_{2}\text{]}}{\text{d}t}=-k_{1}\text{[G}^{\text{a}}\text{][PIP}_{2}\text{]}-k_{2}\text{[PIP}_{2}\text{]}-k_{2}\text{[IP}_{3}\text{]+}k_{2}\text{[PIP}_{2}\text{]}_{\text{tot}} \\ -(\frac{k_{af2p1}}{1+a_{\text{PI3K}}}[1+a_{\text{PI3K}}\tanh(\frac{\pi\tau_{\text{pss}}\text{(}x,y,t\text{)}}{\delta_{Th\text{r}}})e^{-\eta t}]+k_{af2p2})[\text{PIP}_{2}]+k_{ar2}[\text{PIP}_{3}] \end{matrix}$ (S3)

$\frac{\text{d[PIP}_{3}\text{]}}{\text{d}t}=(\frac{k_{af2p1}}{1+a_{\text{PI3K}}}[1+a_{\text{PI3K}}\tanh(\frac{\pi\tau_{\text{pss}}\text{(}x,y,t\text{)}}{\delta_{Th\text{r}}})e^{-\eta t}]+k_{af2p2})[\text{PIP}_{2}]-k_{ar2}[\text{PIP}_{3}]$ (S4)

$\begin{matrix} \frac{\text{d}[\text{Ca}^{2+}]_{\text{c}}}{\text{d}t}=k_{rel}\left(\frac{[\text{IP}_{3}]}{K_{\text{IP}_{3}}+[\text{IP}_{3}]}\right)^{3}[\text{Ca}^{\text{2+}}]_{\text{s}}-k_{res}\left(\frac{[\text{Ca}^{2+}]_{\text{c}}}{K_{\text{Cac\_res}}+[\text{Ca}^{2+}]_{\text{c}}}\right)^{2} \\ +k_{ar3}\text{[Ca}^{\text{2+}}\text{]}_{\text{b}}-k_{af3}(B_{\text{tot}}-\text{[Ca}^{\text{2+}}\text{]}_{\text{b}})\text{[Ca}^{\text{2+}}\text{]}_{\text{c}} \\ +k_{CCE}(1.00-\xi[\text{cGMP}])(\text{[Ca}_{0}^{\text{2+}}\text{]}_{\text{s}}-\text{[Ca}^{\text{2+}}\text{]}_{\text{s}})(\text{[Ca}^{\text{2+}}\text{]}_{\text{e}}-\text{[Ca}^{\text{2+}}\text{]}_{\text{c}}) \\ +\frac{q_{\max}}{1+N\exp(-W_{0}\frac{(\tau_{\text{pss}}\text{(}x,y,t\text{)}+\sqrt{16\chi^{2}+\tau_{\text{pss}}\text{(}x,y,t\text{)}^{2}}-4\chi)^{2}}{\tau_{\text{pss}}\text{(}x,y,t\text{)}+\sqrt{16\chi^{2}+\tau_{\text{pss}}\text{(}x,y,t\text{)}^{2}}})} \\ -k_{out}\left(\frac{[\text{Ca}^{2+}]_{\text{c}}}{K_{\text{Cac\_out}}+[\text{Ca}^{2+}]_{\text{c}}}\right)+k_{leak}[\text{Ca}^{2+}]_{\text{s}}^{2} \end{matrix}$ (S5)$\frac{\text{d}[\text{Ca}^{2+}]_{\text{b}}}{\text{d}t}=k_{af3}(B_{\text{tot}}-\text{[Ca}^{\text{2+}}\text{]}_{\text{b}})\text{[Ca}^{\text{2+}}\text{]}_{\text{c}}-k_{ar3}\text{[Ca}^{\text{2+}}\text{]}_{\text{b}}$ (S6)

$\frac{\text{d}[\text{Ca}^{\text{2+}}]_{\text{s}}}{\text{d}t}=V_{r}(k_{res}\left(\frac{[\text{Ca}^{2+}]_{\text{c}}}{K_{\text{Cac\_res}}+[\text{Ca}^{2+}]_{\text{c}}}\right)^{2}-k_{rel}\left(\frac{[\text{IP}_{3}]}{K_{\text{IP}_{3}}+[\text{IP}_{3}]}\right)^{3}[\text{Ca}^{\text{2+}}]_{\text{s}}-k_{leak}[\text{Ca}^{\text{2+}}\text{]}_{\text{s}}^{2})$ (S7)

$\frac{\text{d}[\text{Ca}_{4}\text{-CaM}]}{\text{d}t}=k_{\text{Ca}_{4}\text{-CaM}}\left(\theta\frac{[\text{Ca}^{\text{2+}}]_{\text{c}}^{\lambda}}{K_{\text{dCa}_{4}\text{-CaM}}+\text{[Ca}^{\text{2+}}\text{]}_{\text{c}}^{\lambda}}[\text{CaM]}_{\text{tot}}-[\text{Ca}_{4}\text{-CaM}]\right)$ (S8)

$\frac{\text{d[PKC}^{\text{p}}\text{]}}{\text{d}t}=k_{af4}\frac{[\text{PIP}_{3}]-[\text{PIP}_{3}]_{\min}}{[\text{PIP}_{3}]_{\max}-[\text{PIP}_{3}]_{\min}}([\text{PKC]}_{\text{tot}}-\text{[PKC}^{\text{p}}])-k_{ar4}[\text{PKC}^{\text{p}}]$ (S9)

$\frac{\text{d[AKT}^{\text{p}}\text{]}}{\text{d}t}=k_{af5}\frac{[\text{PIP}_{3}]-[\text{PIP}_{3}]_{\min}}{[\text{PIP}_{3}]_{\max}-[\text{PIP}_{3}]_{\min}}([\text{AKT]}_{\text{tot}}-[\text{AKT}^{\text{p}}])-k_{ar5}[\text{AKT}^{\text{p}}]$ (S10)

$\begin{matrix} \frac{\text{d}[\text{eNOS-CaM}]}{\text{d}t}=k_{af6}\frac{\text{[Ca}_{4}\text{-CaM]}}{K_{\text{Ca}_{4}\text{-CaM}}\text{+[Ca}_{4}\text{-CaM]}}[\text{eNOS}_{\text{cav}}]-k_{ar6}[\text{eNOS-CaM}] \\ -k_{af7}\frac{\text{[AKT}^{\text{p}}\text{]}}{\text{[AKT]}_{\text{tot}}}[\text{eNOS-CaM}]+k_{ar7}[\text{eNOS-CaM}^{\text{p}}] \end{matrix}$ (S11)

$\frac{\text{d}[\text{eNOS-CaM}^{\text{p}}]}{\text{d}t}=k_{af7}\frac{\text{[AKT}^{\text{p}}\text{]}}{\text{[AKT]}_{\text{tot}}}[\text{eNOS-CaM}]-k_{ar7}[\text{eNOS-CaM}^{\text{p}}]$ (S12)

$\frac{\text{d}[\text{eNOS}_{\text{cav}}^{\text{p+in}}]}{\text{d}t}=k_{af8}\frac{\text{[PKC}^{\text{p}}\text{]}}{\text{[PKC]}_{\text{tot}}}[\text{eNOS}_{\text{cav}}]-k_{ar8}[\text{eNOS}_{\text{cav}}^{\text{p+in}}]$ (S13)

$\begin{matrix} \frac{\text{d}[\text{eNOS}_{\text{cav}}]}{\text{d}t}=-(k_{af6}\frac{\text{[Ca}_{4}\text{-CaM]}}{K_{\text{Ca}_{4}\text{-CaM}}\text{+[Ca}_{4}\text{-CaM]}}[\text{eNOS}_{\text{cav}}]-k_{ar6}[\text{eNOS-CaM}]) \\ -(k_{af8}\frac{\text{[PKC}^{\text{p}}\text{]}}{\text{[PKC]}_{\text{tot}}}[\text{eNOS}_{\text{cav}}]-k_{ar8}[\text{eNOS}_{\text{cav}}^{\text{p+in}}]) \end{matrix}$ (S14)

$\begin{matrix} \frac{\text{d[NO]}}{\text{d}t}=k_{\text{NO}}([\text{eNOS-CaM}]+\mu[\text{eNOS-CaM}^{\text{p}}]) \\ -R_{\text{NO}}^{\max}\frac{[\text{NO}]^{2}+b_{1}[\text{NO}]}{a_{0}+a_{1}[\text{NO}]+[\text{NO}]^{2}}[\text{sGC}]-k_{d\text{NO}}[\text{NO}] \end{matrix}$ (S15)

$\frac{\text{d[cGMP]}}{\text{d}t}=V_{\text{cGMP}}^{\max}\frac{g_{0}+g_{1}[\text{NO}]+[\text{NO}]^{2}}{a_{0}+a_{1}[\text{NO}]+[\text{NO}]^{2}}-\frac{X[\text{cGMP}]^{2}}{K_{\text{cGMP}}+[\text{cGMP}]}$ (S16)
