# Supplementary material for: Multiscale modeling reveals non-linear dynamics of AKT-mediated NO-ROS crosstalk in shear stress-induced endothelial phenotype
Source: PLoS Comput Biol. 2026 Oct 21;22(10):e1014860. doi: 10.1371/journal.pcbi.1014860 (PMC13705609; doi:10.1371/journal.pcbi.1014860)
Supplement: Supplementary file 2 [file pcbi.1014860.s011.docx]

**Responses to Reviewers’ Comments**

We would like to thank all editors and reviewers for their comments and revisions contributed to our manuscript No.: PCOMPBIOL-D-26-01256.

We are very grateful to the editor for providing us the opportunity to revise the manuscript again. All comments proposed by the editors and reviewers have been carefully considered. These comments are really helpful to improve our work. We have added our in-depth discussion and enriched corresponding references to explicitly state details corresponding to all comments.

The detailed responses to reviewers’ comments are listed in the following.

**Reviewer #1**

**Comment 1:** Several biological descriptions are not sufficiently precise and need revised using standard terminology. For example, the descriptions of AKT phosphorylation should confirm the precise site of amino acid‌, oscillatory shear stress (OSS) should be OSS = 0.5(?) ± 5 dyn/cm^2^, ROS-producing cells should be made consistent with current biological conventions and with the assumptions adopted in the mathematical model.

**Response 1:** Thank you very much for this important and constructive comment. We have carefully revised the manuscript with respect to AKT phosphorylation, the definition of oscillatory shear stress (OSS), and the cellular sources of ROS. Our point-by-point responses are provided below.

(1) Clarification of the AKT phosphorylation site

We apologize for the imprecise description of “AKT phosphorylation” in the original manuscript. Based on the relevant literature, Kong et al. reported that shear stress can induce Akt activation through phosphorylation at Thr308/Ser473^[R1]^. Activated AKT subsequently promotes eNOS phosphorylation at Ser-1197, thereby enhancing NO production in endothelial cells^[R2]^. Accordingly, we have clarified the specific phosphorylation sites involved in AKT activation and its downstream regulation of eNOS-mediated NO production. The corresponding description has been revised at **Lines 210-214** of the revised manuscript.

References

[R1] Kong X, Qu X, Li B, et al. Modulation of low shear stress-induced eNOS multi-site phosphorylation and nitric oxide production via protein kinase and ERK1/2 signaling[J]. Molecular medicine reports, 2017, 15(2): 908-914.

[R2] Sriram K, Laughlin J G, Rangamani P, et al. Shear-induced nitric oxide production by endothelial cells[J]. Biophysical journal, 2016, 111(1): 208-221.

(2) Clarification of the definition and magnitude of OSS

Thank you for this important comment. We agree that the definition of OSS should be described more clearly. In the revised manuscript, we have clarified that *τ*(*x*,*y*) represents the spatially varying baseline WSS magnitude, which determines the local OSS amplitude. The instantaneous OSS is defined as

$$\tau_{\text{oss}}\text{(}x,y,t\text{)}=\tau(x,y)\cos(2\pi ft)$$

where the cosine term introduces periodic reversal of the shear-stress direction. Therefore, the values of 5-15 dyn/cm^2^ in our model represent the local OSS amplitudes, rather than mean shear-stress values.

Accordingly, we have revised the relevant descriptions in the manuscript to avoid ambiguity and to make the OSS definition consistent throughout the text. The corresponding changes have been made at **Lines 232, 236–237, 242–244, 407, and 520** in the revised manuscript.

(3) Standardization of the description of ROS-producing cells and consistency with the mathematical model

We apologize for the unclear description of ROS-producing cells in the original manuscript. In the present model, only different endothelial phenotypes and macrophages are explicitly represented. Based on the relevant literature, activated endothelial cells and pro-inflammatory M1 macrophages are recognized as important sources of ROS^[R1,R2]^. Therefore, we defined these two cell populations as the major cellular ROS sources considered in the model.

To avoid misunderstanding, we have revised the corresponding description in Section 2.4 and clarified that activated endothelial cells and M1 macrophages are the ROS-producing cell populations explicitly considered in the current mathematical model. The relevant changes have been made on **Lines 260-265** and **Lines 275-278** of the revised manuscript.

References

[R1] Yan R, Zhang X, Xu W, et al. ROS-induced endothelial dysfunction in the pathogenesis of atherosclerosis[J]. Aging and disease, 2024, 16(1): 250.

[R2] Canton M, Sánchez-Rodríguez R, Spera I, et al. Reactive oxygen species in macrophages: sources and targets[J]. Frontiers in immunology, 2021, 12: 734229.

**Comment 2:** The presentation of several results could be further clarified. Although the Results section is generally well organized, several descriptions could be refined to improve clarity and ensure that the interpretation of the simulation results is unambiguous.

**Response 2:** Thank you for this helpful comment. In the revised manuscript, we have carefully refined the presentation of the simulation results to distinguish direct model observations from their interpretation. We also clarified the definitions of key variables, the timing of phenotypic transitions, and the comparisons among different OSS conditions, AKT treatment times, and AKT activation strengths. In addition, several ambiguous or overly general statements were replaced with more quantitative descriptions directly supported by the corresponding figures. These revisions were made throughout Sections 3.1-3.5 to improve clarity and avoid ambiguous interpretation of the simulation results. The relevant changes have been made at **Lines 438-440, 447-490, 457-462, 597-598, 604-606, 621-622, 692-694, 715-716, and 723-726** of the revised manuscript.

**Comment 3:** The mechanistic interpretation of several simulation results could be expanded. Further discussion of the underlying biological mechanisms would improve the interpretation of the model predictions and their biological relevance.

**Response 3:** Thank you for this valuable comment. We agree that a more detailed mechanistic interpretation would improve the biological relevance of the simulation results. In the revised manuscript, we have expanded the Discussion to further clarify the mechanistic links among AKT activation, eNOS-mediated NO production, ROS accumulation, endothelial phenotypic recovery, and inflammatory regulation. We also refined the interpretation of the effects of AKT treatment timing and activation strength on endothelial repair. During this revision, several repetitive or overly descriptive statements were removed to make the Discussion more concise and precise. These changes improve both the mechanistic depth and the clarity of the biological interpretation of the model predictions. The relevant revisions have been made at **Lines 785-791, 792-793, 800-801, 816-819, 822-825, and 836-845** of the revised manuscript.

**Comment 4**: The manuscript investigates hemodynamic properties. Please add mechanical information, such as the direction of blood flow, to key parameters (e.g., Figure 2).

**Response 4:** Thank you for this helpful comment. We agree that the mechanical information should be described more clearly. In the present model, the blood-flow velocity field and flow direction are not explicitly resolved. Instead, wall shear stress is prescribed as an external mechanical input at each spatial position, and the model focuses on the spatially dependent endothelial responses to different local shear-stress conditions. To avoid ambiguity, we have clarified this modeling assumption in Section 2.3 and in the corresponding figure description. These revisions make the mechanical loading conditions more explicit without changing the model formulation or simulation results. The relevant changes have been made at **Lines** **228-231, 245-248, and 438-440** of the revised manuscript.

**Reviewer #2**

**Comment 1:** The computational domain is described inconsistently.

Lines 223-232 state that the domain is 400×400×1 pixels with a pixel size of 2 μm, but the reported physical size is 0.8×0.2 mm, while Eq. (8) uses x∈[0,400] and y∈[0,100]. The authors should verify the actual lattice size and correct the domain dimensions, coordinates, and related unit conversions.

**Response 1:** Thank you for identifying this inconsistency. We carefully rechecked the computational domain and the corresponding coordinate definitions. The correct lattice size used in the simulations is 400×100×1 pixels, with each pixel corresponding to a physical length of 2 μm. Therefore, the computational domain corresponds to 0.8×0.2 mm, consistent with the spatial ranges used in Eq. (8), x∈[0,400] and y∈[0,100] in lattice coordinates. The previously reported value of 400×400×1 pixels was a typographical error. We have corrected the computational-domain description and carefully checked the related dimensions, coordinates, and unit conversions throughout the revised manuscript to ensure consistency. These corrections are purely descriptive and do not affect the model implementation or simulation results. The relevant correction has been made at **Line 225** of the revised manuscript.

**Comment 2:** The AKT treatment strength and the resulting conclusions should be clarified.

Lines 381-386 describe AKT activation as 2-5-fold changes, whereas later sections appear to use different numerical values. The exact meaning and units of kaf5 should be standardized throughout the text, figures, and supplementary materials. In addition, claims regarding an optimal or excessive AKT activation level should be weakened unless they are supported by a clearer quantitative criterion and consistent parameter analysis.

**Response 2:** Thank you for this important comment. We agree that the definition and interpretation of AKT activation strength should be standardized and stated more cautiously. In the revised manuscript, we clarified the role of *k*_af5_ as the reference parameter for AKT activation strength and explicitly defined the three representative activation levels used in the multiscale repair simulations as 3.4 *k*_af5_ (weak activation), 3.8 *k*_af5_ (moderate activation), and 4.2 *k*_af5_ (strong activation). We also standardized these descriptions throughout the relevant Results and figure captions. In addition, we revised the interpretation of the activation-strength analysis to avoid overstatement. Rather than referring to 3.8 *k*_af5_ as an “optimal” level or 4.2 *k*_af5_ as “excessive” activation, we now state that among the three tested activation strengths, 3.8 *k*_af5_ showed the most balanced combination of QEC recovery, increased NO, and reduced ROS and MCP-1, whereas 4.2 *k*_af5_ produced the fastest AEC-to-QEC transition and the greatest reduction in ROS and MCP-1. These revisions improve the consistency and precision of the parameter description and ensure that the conclusions remain within the tested parameter range. The relevant changes have been made at **Lines 399-405** and **676-677** of the revised manuscript.

**Comment 3:** Section numbering is incorrect.

Two subsections are numbered 2.5. The section “Monocyte Recruitment, Polarization, and Chemotaxis” and all subsequent sections should be renumbered.

**Response 3:** Thank you for pointing out this numbering error. We have corrected the duplicate subsection numbering in Section 2. Specifically, the subsection “Monocyte Recruitment, Polarization, and Chemotaxis” and all subsequent subsections have been renumbered sequentially to ensure consistency throughout the manuscript. The corresponding section numbers and related cross-references have also been updated accordingly. The relevant corrections have been made at **Lines 331 and 373** of the revised manuscript.

**Comment 4:** The OSS terminology is unclear.

Lines 228-238 define OSS as a zero-mean sinusoidal function, but the manuscript later uses terms such as “low OSS” and “LOSS.” The authors should clarify whether 5, 10, and 15 dyn/cm2 represent OSS amplitudes and use consistent terminology throughout.

**Response 4:** Thank you for pointing out this ambiguity. We agree that the OSS terminology should be defined and used more consistently. In the revised manuscript, we clarified that the values of 5, 10, and 15 dyn/cm² represent local OSS amplitudes, rather than temporal mean shear-stress values. We also standardized the related terminology throughout the manuscript by replacing ambiguous expressions such as “low OSS,” “LOSS,” and “OSS levels” with more precise descriptions, including “lower local OSS amplitude” and “local OSS amplitudes of 5, 10, and 15 dyn/cm^2^,” where appropriate. The relevant clarification has been made at Lines **242-248, 520 and 530** of the revised manuscript.

**Comment 5:** The interpretation of NO dynamics is inconsistent.

Lines 433-438 report that total NO concentration continues to increase, but this is then interpreted as progressively limited NO synthesis. The authors should distinguish between total NO concentration, local NO bioavailability, NO production rate, and ROS-mediated NO depletion.

**Response 5:** Thank you for pointing out this inconsistency. We agree that the previous interpretation did not clearly distinguish total NO concentration from NO production and local NO bioavailability. In the revised manuscript, we have clarified these concepts and revised the corresponding Results description. Specifically, the simulation shows that total NO concentration continues to increase over time, although its rate of increase becomes slower at later stages, while ROS accumulates progressively. We therefore no longer interpret this trend as a progressive limitation of NO synthesis. Instead, we clarify that increased ROS enhances NO consumption and may reduce local NO bioavailability despite the continued increase in total NO concentration, thereby contributing to a progressively more oxidative microenvironment. The relevant changes have been made at **Lines 457-462** of the revised manuscript.

**Comment 6:** The macrophage-polarization conclusion is overstated.

Lines 337-343 prescribe a fixed M1:M2 differentiation ratio of 2:1. Therefore, the model cannot independently demonstrate that AKT has little effect on macrophage polarization. The conclusion should be revised to reflect that the ratio is predefined by the model.

**Response 6:** Thank you for this important comment. In the current model, the M1:M2 differentiation ratio is predefined as 2:1. Therefore, the model does not independently evaluate AKT-dependent changes in macrophage polarization preference. We have clarified this modeling assumption in the Methods and revised the corresponding Discussion and major conclusion. The revised manuscript now emphasizes that AKT can still indirectly influence the abundance of M1 and M2 macrophages by altering endothelial redox homeostasis, MCP-1 signaling, and monocyte recruitment, whereas direct regulation of the M1:M2 polarization ratio is not independently resolved in the present model. The relevant changes have been made at **Lines 357-359, 837-845, and 869-872** of the revised manuscript.

**Comment 7:** Several equations and symbols require correction.

All equations should be checked against the model implementation and reformatted for readability.

**Response 7:** Thank you for this careful comment. We systematically rechecked all equations and symbols against the model implementation. Most equations were confirmed to be consistent with the implemented model. We identified a presentation error in Eq. (S1), where the condition for endothelial damage accumulation was written incorrectly.

$\Delta D=\left\{\begin{matrix} \text{0, }C_{\text{cNO}}\geq D_{\text{NOthr}} \\ \frac{V_{\text{NO-}\max}C_{\text{cNO}}^{n}}{C_{\text{cNO}}^{n}+K_{\text{NO}}^{n}}-\frac{V_{\text{NO-}\max}D_{\text{NO}}^{n}}{D_{\text{NO}}^{n}+K_{\text{NO}}^{n}}\text{, }C_{\text{cNO}}<D_{\text{NOthr}}\text{ } \end{matrix}\right.$ (S1)

This equation has now been corrected to indicate that damage accumulates when the local NO concentration falls below the physiological threshold. We also reviewed the formatting and notation of the remaining equations to improve readability and consistency. The corresponding condition was correctly implemented in the simulation code; therefore, this correction does not affect the model implementation or simulation results. The relevant correction has been made at **Line** **308** of the revised manuscript.
