# Supplementary material for: Multiscale modeling reveals non-linear dynamics of AKT-mediated NO-ROS crosstalk in shear stress-induced endothelial phenotype
Source: PLoS Comput Biol. 2026 Oct 21;22(10):e1014860. doi: 10.1371/journal.pcbi.1014860 (PMC13705609; doi:10.1371/journal.pcbi.1014860)
Supplement: Peer review history

# Peer review: Decision Letter 0

**DOI:** 10.1371/journal.pcbi.1014860.r001

**Authors:** Xiaoqiang Sun, Stacey D. Finley

1 Sep 2026

PCOMPBIOL-D-26-01256

Multiscale modeling reveals non-linear dynamics of AKT-mediated NO-ROS crosstalk in shear stress-induced endothelial phenotype

PLOS Computational Biology

Dear Dr. Qin,

Thank you for submitting your manuscript to PLOS Computational Biology. After careful consideration, we feel that it has merit but does not fully meet PLOS Computational Biology's publication criteria as it currently stands. Therefore, we invite you to submit a revised version of the manuscript that addresses the points raised during the review process.

Please submit your revised manuscript by Oct 31 2026 11:59PM. If you will need more time than this to complete your revisions, please reply to this message or contact the journal office at ploscompbiol@plos.org. When you're ready to submit your revision, log on to https://www.editorialmanager.com/pcompbiol/ and select the 'Submissions Needing Revision' folder to locate your manuscript file.

Please include the following items when submitting your revised manuscript:

* A letter that responds to each point raised by the editor and reviewer(s). You should upload this letter as a separate file labeled 'Response to Reviewers'. This file does not need to include responses to formatting updates and technical items listed in the 'Journal Requirements' section below.

* A marked-up copy of your manuscript that highlights changes made to the original version. You should upload this as a separate file labeled 'Revised Manuscript with Track Changes'.

* An unmarked version of your revised paper without tracked changes. You should upload this as a separate file labeled 'Manuscript'.

If you would like to make changes to your financial disclosure, competing interests statement, or data availability statement, please make these updates within the submission form at the time of resubmission. Guidelines for resubmitting your figure files are available below the reviewer comments at the end of this letter. As the corresponding author, your ORCID iD is verified in the submission system and will appear in the published article. PLOS supports the use of ORCID, and we encourage all coauthors to register for an ORCID iD and use it as well. Please encourage your coauthors to verify their ORCID iD within the submission system before final acceptance, as unverified ORCID iDs will not appear in the published article. Only the individual author can complete the verification step; PLOS staff cannot verify ORCID iDs on behalf of authors.

We look forward to receiving your revised manuscript.

Kind regards,

Xiaoqiang Sun

Academic Editor

PLOS Computational Biology

Stacey Finley

Section Editor

PLOS Computational Biology

**Journal Requirements:**

If the reviewer comments include a recommendation to cite specific previously published works, please review and evaluate these publications to determine whether they are relevant and should be cited. There is no requirement to cite these works unless the editor has indicated otherwise.

1) We ask that a manuscript source file is provided at Revision. Please upload your manuscript file as a .doc, .docx, .rtf or .tex. If you are providing a .tex file, please upload it under the item type u2018LaTeX Source Fileu2019 and leave your .pdf version as the item type u2018Manuscriptu2019.

2) Please upload all main figures as separate Figure files in .tif or .eps format. For more information about how to convert and format your figure files please see our guidelines:

https://journals.plos.org/ploscompbiol/s/figures

3) Please upload a copy of Figure Fig 1-10 which you refer to in your text on pages 4, 12, 13, 14, 15, 16, 18, 20, 21, 22, and 25. Or, if the figure is no longer to be included as part of the submission please remove all reference to it within the text.

4) Please amend your detailed Financial Disclosure statement. This is published with the article. It must therefore be completed in full sentences and contain the exact wording you wish to be published.

i) State the initials, alongside each funding source, of each author to receive each grant. For example: "This work was supported by the National Institutes of Health (####### to AM; ###### to CJ) and the National Science Foundation (###### to AM)."

ii) State what role the funders took in the study. If the funders had no role in your study, please state: "The funders had no role in study design, data collection and analysis, decision to publish, or preparation of the manuscript."

iii) If any authors received a salary from any of your funders, please state which authors and which funders..

If you did not receive any funding for this study, please simply state: u201cThe authors received no specific funding for this work.u201d

5) We have amended your Competing Interest statement to comply with journal style. We kindly ask that you double check the statement and let us know if anything is incorrect.

**Reviewers' comments:**

Reviewer's Responses to Questions

**Comments to the Authors:**

**Please note here if the review is uploaded as an attachment.**

Reviewer #1: The manuscript presents a multiscale computational framework integrating hemodynamic stimulation, intracellular AKT signaling, eNOS/NO, oxidative stress, and endothelial repair. The study addresses an important scientific question regarding the mechanisms underlying endothelial injury and repair under disturbed flow conditions. The mathematical framework is well designed. The biological mechanisms are reasonably integrated. Overall, the work demonstrates good scientific significance and methodological innovation and provides valuable mechanistic insights into AKT-mediated endothelial repair.

1. Several biological descriptions are not sufficiently precise and need revised using standard terminology. For example, the descriptions of AKT phosphorylation should confirm the precise site of amino acid舄, oscillatory shear stress (OSS) should be OSS = 0.5(?) ± 5 dyn/cm2, ROS-producing cells should be made consistent with current biological conventions and with the assumptions adopted in the mathematical model.

2. The presentation of several results could be further clarified. Although the Results section is generally well organized, several descriptions could be refined to improve clarity and ensure that the interpretation of the simulation results is unambiguous.

3. The mechanistic interpretation of several simulation results could be expanded. Further discussion of the underlying biological mechanisms would improve the interpretation of the model predictions and their biological relevance.

4. The manuscript investigates hemodynamic properties. Please add mechanical information, such as the direction of blood flow, to key parameters (e.g., Figure 2).

Reviewer #2: The manuscript presents a multiscale ODE-PDE-CPM framework for investigating AKT-mediated NO-ROS crosstalk during oscillatory shear stress-induced endothelial injury and repair. The topic is relevant, and the integration of intracellular signaling, extracellular biochemical transport, endothelial phenotypic transitions, and immune-cell dynamics is potentially valuable. However, the manuscript currently contains several inconsistencies in the model description, numerical settings, figure references, and interpretation of the simulation results. Therefore, this paper needs to be revised accordingly before it can be considered for publication.

Comment 1. The computational domain is described inconsistently.

Lines 223-232 state that the domain is 400×400×1 pixels with a pixel size of 2 μm, but the reported physical size is 0.8×0.2 mm, while Eq. (8) uses x∈[0,400] and y∈[0,100]. The authors should verify the actual lattice size and correct the domain dimensions, coordinates, and related unit conversions.

Comment 2. The AKT treatment strength and the resulting conclusions should be clarified.

Lines 381-386 describe AKT activation as 2-5-fold changes, whereas later sections appear to use different numerical values. The exact meaning and units of kaf5 should be standardized throughout the text, figures, and supplementary materials. In addition, claims regarding an optimal or excessive AKT activation level should be weakened unless they are supported by a clearer quantitative criterion and consistent parameter analysis.

Comment 3. Section numbering is incorrect.

Two subsections are numbered 2.5. The section “Monocyte Recruitment, Polarization, and Chemotaxis” and all subsequent sections should be renumbered.

Comment 4. The OSS terminology is unclear.

Lines 228-238 define OSS as a zero-mean sinusoidal function, but the manuscript later uses terms such as “low OSS” and “LOSS.” The authors should clarify whether 5, 10, and 15 dyn/cm2 represent OSS amplitudes and use consistent terminology throughout.

Comment 5. The interpretation of NO dynamics is inconsistent.

Lines 433-438 report that total NO concentration continues to increase, but this is then interpreted as progressively limited NO synthesis. The authors should distinguish between total NO concentration, local NO bioavailability, NO production rate, and ROS-mediated NO depletion.

Comment 6. The macrophage-polarization conclusion is overstated.

Lines 337-343 prescribe a fixed M1:M2 differentiation ratio of 2:1. Therefore, the model cannot independently demonstrate that AKT has little effect on macrophage polarization. The conclusion should be revised to reflect that the ratio is predefined by the model.

Comment 7. Several equations and symbols require correction.

All equations should be checked against the model implementation and reformatted for readability.

**********

**Have the authors made all data and (if applicable) computational code underlying the findings in their manuscript fully available?**

The PLOS Data policy requires authors to make all data and code underlying the findings described in their manuscript fully available without restriction, with rare exception (please refer to the Data Availability Statement in the manuscript PDF file). The data and code should be provided as part of the manuscript or its supporting information, or deposited to a public repository. For example, in addition to summary statistics, the data points behind means, medians and variance measures should be available. If there are restrictions on publicly sharing data or code —e.g. participant privacy or use of data from a third party—those must be specified.

Reviewer #1: Yes

Reviewer #2: Yes

**********

PLOS authors have the option to publish the peer review history of their article (what does this mean?). If published, this will include your full peer review and any attached files.

If you choose “no”, your identity will remain anonymous but your review may still be made public.

**Do you want your identity to be public for this peer review?**  For information about this choice, including consent withdrawal, please see our Privacy Policy.

Reviewer #1: No

Reviewer #2: No

[NOTE: If reviewer comments were submitted as an attachment file, they will be attached to this email and accessible via the submission site. Please log into your account, locate the manuscript record, and check for the action link "View Attachments". If this link does not appear, there are no attachment files.]

**Figure resubmission:**

While revising your submission, we strongly recommend that you use PLOS’s NAAS tool (https://ngplosjournals.pagemajik.ai/artanalysis) to test your figure files. NAAS can convert your figure files to the TIFF file type and meet basic requirements (such as print size, resolution), or provide you with a report on issues that do not meet our requirements and that NAAS cannot fix.

After uploading your figures to PLOS’s NAAS tool - https://ngplosjournals.pagemajik.ai/artanalysis, NAAS will process the files provided and display the results in the "Uploaded Files" section of the page as the processing is complete. If the uploaded figures meet our requirements (or NAAS is able to fix the files to meet our requirements), the figure will be marked as "fixed" above. If NAAS is unable to fix the files, a red "failed" label will appear above. When NAAS has confirmed that the figure files meet our requirements, please download the file via the download option, and include these NAAS processed figure files when submitting your revised manuscript.

**Reproducibility:**

To enhance the reproducibility of your results, we recommend that authors of applicable studies deposit laboratory protocols in protocols.io, where a protocol can be assigned its own identifier (DOI) such that it can be cited independently in the future. Additionally, PLOS ONE offers an option to publish peer-reviewed clinical study protocols. Read more information on sharing protocols at https://plos.org/protocols?utm_medium=editorial-email&utm_source=authorletters&utm_campaign=protocols

# Author comment: Author response to Decision Letter 1

**DOI:** 10.1371/journal.pcbi.1014860.r002

12 Sep 2026

Attachment Submitted filename: Response to Reviewers-PCOMPBIOL-D-26-01256.docx

# Peer review: Decision Letter 1

**DOI:** 10.1371/journal.pcbi.1014860.r003

**Authors:** Xiaoqiang Sun, Stacey D. Finley

27 Sep 2026

Dear Professor Qin,

We are pleased to inform you that your manuscript 'Multiscale modeling reveals non-linear dynamics of AKT-mediated NO-ROS crosstalk in shear stress-induced endothelial phenotype' has been provisionally accepted for publication in PLOS Computational Biology.

Before your manuscript can be formally accepted you will need to complete some formatting changes, which you will receive in a follow up email. A member of our team will be in touch with a set of requests.

Please note that your manuscript will not be scheduled for publication until you have made the required changes, so a swift response is appreciated.

IMPORTANT: The editorial review process is now complete. PLOS will only permit corrections to spelling, formatting or significant scientific errors from this point onwards. Requests for major changes, or any which affect the scientific understanding of your work, will cause delays to the publication date of your manuscript.

Should you, your institution's press office or the journal office choose to press release your paper, you will automatically be opted out of early publication. We ask that you notify us now if you or your institution is planning to press release the article. All press must be co-ordinated with PLOS.

Thank you again for supporting Open Access publishing; we are looking forward to publishing your work in PLOS Computational Biology.

Best regards,

Xiaoqiang Sun

Academic Editor

PLOS Computational Biology

Stacey Finley

Section Editor

PLOS Computational Biology

***********************************************************

Reviewer's Responses to Questions

**Comments to the Authors:**

**Please note here if the review is uploaded as an attachment.**

Reviewer #1: accepted

Reviewer #2: The authors have revised the manuscript very carefully and I have no more comments.

**********

**Have the authors made all data and (if applicable) computational code underlying the findings in their manuscript fully available?**

The PLOS Data policy requires authors to make all data and code underlying the findings described in their manuscript fully available without restriction, with rare exception (please refer to the Data Availability Statement in the manuscript PDF file). The data and code should be provided as part of the manuscript or its supporting information, or deposited to a public repository. For example, in addition to summary statistics, the data points behind means, medians and variance measures should be available. If there are restrictions on publicly sharing data or code —e.g. participant privacy or use of data from a third party—those must be specified.

Reviewer #1: Yes

Reviewer #2: Yes

**********

PLOS authors have the option to publish the peer review history of their article (what does this mean?). If published, this will include your full peer review and any attached files.

If you choose “no”, your identity will remain anonymous but your review may still be made public.

**Do you want your identity to be public for this peer review?**  For information about this choice, including consent withdrawal, please see our Privacy Policy.

Reviewer #1: **Yes:**  Juhui Qiu

Reviewer #2: No

# Editor report: Acceptance letter

**DOI:** 10.1371/journal.pcbi.1014860.r004

**Authors:** Xiaoqiang Sun, Stacey D. Finley

PCOMPBIOL-D-26-01256R1

Multiscale modeling reveals non-linear dynamics of AKT-mediated NO-ROS crosstalk in shear stress-induced endothelial phenotype

Dear Dr Qin,

I am pleased to inform you that your manuscript has been formally accepted for publication in PLOS Computational Biology. Your manuscript is now with our production department and you will be notified of the publication date in due course.

The corresponding author will soon be receiving a typeset proof for review, to ensure errors have not been introduced during production. Please review the PDF proof of your manuscript carefully, as this is the last chance to correct any errors. Please note that major changes, or those which affect the scientific understanding of the work, will likely cause delays to the publication date of your manuscript.

Soon after your final files are uploaded, unless you have opted out, the early version of your manuscript will be published online. The date of the early version will be your article's publication date. The final article will be published to the same URL, and all versions of the paper will be accessible to readers.

For Research, Software, and Methods articles, you will receive an invoice from PLOS for your publication fee after your manuscript has reached the completed accept phase. If you receive an email requesting payment before acceptance or for any other service, this may be a phishing scheme. Learn how to identify phishing emails and protect your accounts at https://explore.plos.org/phishing.

Thank you again for supporting PLOS Computational Biology and open-access publishing. We are looking forward to publishing your work!

With kind regards,

Sharmila Kamatchi

PLOS Computational Biology | Carlyle House, Carlyle Road, Cambridge CB4 3DN | United Kingdom ploscompbiol@plos.org | Phone +44 (0) 1223-442824 | ploscompbiol.org | @PLOSCompBiol
